# Overexpression of a Banana Aquaporin Gene *MaPIP1;1* Enhances Tolerance to Multiple Abiotic Stresses in Transgenic Banana and Analysis of Its Interacting Transcription Factors

**DOI:** 10.3389/fpls.2021.699230

**Published:** 2021-08-25

**Authors:** Yi Xu, Juhua Liu, Caihong Jia, Wei Hu, Shun Song, Biyu Xu, Zhiqiang Jin

**Affiliations:** ^1^Key Laboratory of Genetic Improvement of Bananas, Haikou Experimental Station, Chinese Academy of Tropical Agricultural Sciences, Haikou, China; ^2^Key Laboratory of Biology and Genetic Resources of Tropical Crops, Institute of Tropical Bioscience and Biotechnology, Chinese Academy of Tropical Agricultural Sciences, Haikou, China; ^3^Sanya Research Institute of Chinese Academy of Tropical Agricultural Sciences, Sanya, China; ^4^Hainan Key Laboratory for Biosafety Monitoring and Molecular Breeding in Off-Season Reproduction Regions, Sanya, China

**Keywords:** *MaPIP1*, tolerance, banana, interaction, transcription factor

## Abstract

Aquaporins can improve the ability of plants to resist abiotic stresses, but the mechanism is still not completely clear. In this research, overexpression of *MaPIP1;1* in banana improved tolerance to multiple stresses. The transgenic plants resulted in lower ion leakage and malondialdehyde content, while the proline, chlorophyll, soluble sugar, and abscisic acid (ABA) contents were higher. In addition, under high salt and recovery conditions, the content of Na^+^ and K^+^ is higher, also under recovery conditions, the ratio of K^+^/Na^+^ is higher. Finally, under stress conditions, the expression levels of ABA biosynthesis and response genes in the transgenic lines are higher than those of the wild type. In previous studies, we proved that the MaMADS3 could bind to the promoter region of *MaPIP1;1*, thereby regulating the expression of *MaPIP1;1* and affecting the drought tolerance of banana plants. However, the mechanism of *MaPIP1;1* gene response to stress under different adversity conditions might be regulated differently. In this study, we proved that some transcription factor genes, including MaERF14, MaDREB1G, MaMYB1R1, MaERF1/39, MabZIP53, and MaMYB22, showed similar expression patterns with *MaPIP1;1* under salt or cold stresses, and their encoded proteins could bind to the promoter region of *MaPIP1;1*. Here we proposed a novel *MaPIP1;1*-mediated mechanism that enhanced salt and cold tolerance in bananas. The results of this study have enriched the stress-resistant regulatory network of aquaporins genes and are of great significance for the development of molecular breeding strategies for stress-resistant fruit crops.

## Introduction

Drought, salinity, and cold stresses can cause plants to lose water and severely affect their growth and development. Water transport is an important way to maintain tolerance to drought and high salt stresses (Cheeseman, 1988; Bray, 1993; Blumwald, 2000). As the main fruit and crop on a global scale, bananas have made important economic contributions to tropical and subtropical developing countries (Liu et al., 2017). Due to their shallow root system, banana plants are susceptible to drought, salt, and cold stress-induced water shortage conditions, which will greatly reduce the yield and quality of bananas (van Asten et al., 2011; Sreedharan et al., 2013).

Aquaporin can increase the penetration of cell membranes to water, glycerol, carbon dioxide, boron, and other small molecules (Kapilan et al., 2018). The aquaporin (AQP) family was first identified from humans and then was isolated from animals and plants. There are currently 47 AQP members in bananas (Hu et al., 2015) and tomatoes (Reuscher et al., 2013), and 33, 35, 53, and 36 AQP members in rice, *Arabidopsis*, Chinese cabbage, and corn, respectively (Chaumont et al., 2001; Johanson et al., 2001; Sakurai et al., 2005; Tao et al., 2014). Plant AQPs can be divided into eight categories, including tonoplast intrinsic proteins (TIPs), plasma membrane intrinsic proteins (PIPs), small basic intrinsic proteins (SIPs), nodulin 26-like intrinsic proteins (NIPs), hybrid intrinsic proteins (HIPs), GlpF-like intrinsic proteins (GIPs), large intrinsic proteins (LIPs), and uncategorized members designated X intrinsic proteins (XIPs), which were based on protein sequence homology and predicted subcellular location (Hussain et al., 2019).

Many biological studies have shown that AQPs not only participate in plant growth and development also can improve the tolerance of plants to drought, high salt, low temperature, and other abiotic stresses (Hu et al., 2012; Feng et al., 2018, 2019; Madrid-Espinoza et al., 2018; Hussain et al., 2019; Li W. et al., 2019). One of the AQP genes, *TaAQP8* in wheat, can improve the salt tolerance of transgenic tobacco (Hu et al., 2012). Under the high salt stress, tobacco *NtAQP1* can improve plant water absorption capacity (Sade et al., 2010). Overexpressing of *SpAQP1*, *TdPIP1;1*, *OsPIP1;1*, *SlTIP2;2*, and *TaNIP* can improve the salt tolerance of transgenic plants (Gao et al., 2010; Chang et al., 2016). Two AQP genes in rice, *OsPIP1*, and *OsPIP2*, improve the drought resistance of transgenic plants. Some members of the plant AQP family can respond to multiple stresses, such as *BnPIP1* and *VfPIP1* can enhance the drought and osmotic ability of transgenic plants (Lian et al., 2004; Guo et al., 2006). *PgTIP1* confers tolerance to drought and salt stresses. *TaAQP7* transgenic plants can respond to drought and osmotic stresses (Peng et al., 2007; Huang et al., 2014). *MaPIP2-7* improves the adaptability of transgenic bananas to drought, salt, and low temperature (Xu et al., 2020c), and bananas transformed with the *MaSIP2-1* gene had a stronger drought and cold tolerance than the control (Xu et al., 2020b). AQP can improve the tolerance of plants to abiotic stress, so further study of its mechanism is very important to improve plant growth and development.

The mechanism by which AQPs improve stress tolerance is still poorly understood. Related studies have shown that AP2/ERF transcription factors have a certain regulatory effect on the expression of AQPs (Xu et al., 2020b). AtTG, which encodes AP2/ERF transcription factors, improves the adaptability to drought tolerance of plants by directly activating the expression of *AtTIP2;3*, *AtTIP1;1*, and *AtPIP2;2* (Zhu et al., 2014). *RdreB1BI* improves the drought tolerance by combining with the *FvPIP2;1* promoter in the strawberry (Gu et al., 2017). Our previous research also showed that MaMADS3 could interact with the promoter of *MaPIP1;1* and improved the drought resistance of plants (Xu et al., 2020a).

In previous studies, we have cloned *MaPIP1;1* in bananas and proved that it could improve the adaptability of transgenic *Arabidopsis* to drought and salt (Xu et al., 2014). Next, the transcription factors that bind to *MaPIP1;1* under drought conditions were screened by the yeast one-hybrid to further analyze the mechanism of *MaPIP1;1* in improving plant drought resistance (Xu et al., 2020a). Our research also shows that *MaPIP1;1* can improve the drought, salt, and low-temperature tolerance of transgenic bananas. In addition, we identified that MaERF14, MaDREB1G, and MaMYB1R1 could regulate *MaPIP1;1* expression under high salt conditions. Furthermore, MaERF1/39, MabZIP53, and MaMYB22 can combine with the *MaPIP1;1* promoter, thereby regulating the expression of *MaPIP1;1* and affect the cold tolerance of banana plants. The results of this study will enhance our understanding of AQP, improving the tolerance of transgenic bananas to abiotic stress and the mechanism by which *MaPIP1;1* improves plant stress resistance and lays the foundation for the cultivation of stress-resistant bananas.

## Materials and Methods

### Plant Materials and Treatments

Banana seedlings (*Musa acuminata* L. AAA group, cv. Brazilian) were grown in coconut medium in a greenhouse (16 h light/8 h dark cycle; 70% relative humidity; 28°C; 200 μmol m^–2^ s^–1^ light intensity). When the seedlings had five leaves (100 days old), those subjected to the stress treatment were grown in the same way.

Under the NaCl treatment, banana seedlings were treated with a Hoagland solution containing 250 mM NaCl for 2, 4, or 6 h, respectively.

In the low-temperature treatment, the banana seedlings were put in an incubator at 28, 15, 10, 7, or 5°C for 12 h (Xu et al., 2014).

### Genetic Transformation and Characterization of Transgenic Plants

The open reading frame (ORF) of *MaPIP1;1* with an *Nco*I/*Spe*I restriction site was cloned into the pCAMBIA1302 vector under the control of a CaMV35S promoter, the primer pair for construction of recombinant vector was listed in Supplementary Table 1. The pCAMBIA1302-*MaPIP1;1*-GFP construct was introduced into Agrobacterium strain EHA105 and transformed *Agrobacterium*. We transformed *MaPIP1;1* into Gongjiao (*Musa acuminata* L. AA group, cv. Mas) using our previously established *Agrobacterium*-mediated transformation method (Liu et al., 2017). The transformed hygromycin-resistant transgenic lines were detected by PCR amplification of the *GFP* gene. The primer pair for PCR verification was listed in Supplementary Table 2. The transgenic plants with positive results through PCR verification have been obtained and planted in the soil. Among them, two independent individuals with the strongest growth have been chosen through Southern blot analysis that further confirmed the integration of *MaPIP1;1* into line 1 (L1) and line 2 (L2) banana genomes. These two lines were randomly selected and planted in the field to obtain their sucking buds, and through their asexual reproduction of sucking buds, the third-generation tissue culture of more than 100 strains each of L1 and L2 was obtained by vegetative propagation for the next experiments.

### Southern Blot Analyses

Digestion of genomic DNA in transgenic banana leaves was performed with *Hin*dIII restriction enzyme. The digested DNA was separated on a 0.8% agarose gel and transferred to a nylon membrane. Design of 35S promoter primers as probes for Southern blot amplification. The primer pair was listed in Supplementary Table 2. The probe was labeled using a random primer labeling system. Hybridization was carried out according to the instructions for use (Roche11745832910, DIG-High Prime DNA Labeling and Detection Starter Kit, Roche, Indianapolis, IN, United States).

### Drought, Salt, and Cold Tolerance Assays of Transgenic and WT Plants

To test the drought, high salt, and low-temperature tolerance of transgenic plants compared with wild-type (WT) plants, banana seedlings were treated with drought, high salt, and cold stresses. In the drought treatment, banana seedlings (100 days old) were subjected to water control for 10 and 15 days; after 15 days of drought stress, they recovered for 10 days. Banana seedlings (100 days old) used for salt treatment were irrigated with 250 mM NaCl for 13 days and then recovered for 10 days. In the cold treatment, banana seedlings (100 days old) were placed in an 8°C incubator for 5 days and then recovered for 11 days.

### Quantification of IL, MDA, and H_2_O_2_ Contents

The measurement method of ion leakage (IL) is as follows: strips cut from banana leaves were placed in distilled water (15 mL) at 25°C for 12 h, and conductivity (C1) was measured using a conductivity meter (DDBJ-350; INESA Scientific Instrument Co., Shanghai, China). Samples were transferred to boiling water for 30 min. When the sample cooled, it was used to test the conductivity (C2). IL was calculated as follows: IL (%) = C1/C2 × 100. The content of malondialdehyde (MDA) was determined by thiobarbituric acid colorimetry (Heath and Packer, 1968). According to the instructions, a test kit (Nanjing Jiancheng Institute of Biological Engineering, Nanjing, A064) was used to measure the content of H_2_O_2_.

### Measurement of Soluble Sugar, Chlorophyll, Proline, GA, and ABA

The phenol reaction method was used to measure the soluble sugar content in the leaves. Banana leaves were placed in boiling water. After cooling, 9% phenol and concentrated sulfuric acid were added to the extract. The mixed solution was incubated at 25°C for 30 min, and the water extract was measured at a wavelength of 485 nm.

A chlorophyll analyzer (SPAD-502Plus, Konica Minolta, Tokyo, Japan) was used to measure the chlorophyll content. Using an ABA analysis kit (CSBE09159Pl, Cusabio, Wuhan Huamei Biotechnology Co., Ltd., Wuhan, China), we determined the ABA content according to the instructions. The proline content was determined using a PRO measurement kit (BC0290, Solarbio, Beijing, China). The gibberellin content was determined using a GA measurement kit (CK-E91022, Jinkelong, Beijing, China).

### Measurement of Na^+^ and K^+^ Contents

Banana leaves were incubated at 105°C for 8 min and then heated to 80°C for 48 h, after which 50 mg of dried leaves was dissolved in nitric acid (6 mL) and 30% H_2_O_2_ (2 mL), followed by heating at 180°C for 15 min. After digestion, dilution was performed with 50 mL ultrapure water, and atomic absorption spectrometry (Analyst400, PerkinElmer, Waltham, MA, United States) was used to determine the content.

### Quantitative PCR

SYBR^®^Premix Ex real-time qRT-PCR was used to test the expression level of *MaPIP1;1* in banana leaves after various treatments, as well as the ABA biosynthesis and biosynthesis in wild and transgenic lines under drought, high salt, and low-temperature conditions. The primer pair for qRT-PCR, was listed in Supplementary Table 3 and the sequences were in Supplementary Figure 4. To respond to gene expression and determine the expression of genes screened by yeast one-hybrid under high salt and low-temperature conditions, use the Stratagene Mx3000P (Stratagene, La Jolla, CA, United States) instrument with Taq^TM^ (TaKaRa Bio, Shiga, Japan) Reagents. To better amplify the target gene and reference gene, a series of diluted primers and templates are designed to obtain the best concentration of primers and templates. The primer pair was shown in Supplementary Table 5. The amplification efficiency of primers is between 0.89 and 1.12. MaActin1 was used as an internal control to normalize the expression level of target genes. Use 2^–ΔΔCt^ to evaluate the expression level of the tested gene.

### cDNA Library Construction and Y1H Library Screening

The plant RNA kit (TIANGEN Biotech, Beijing, China) was used to extract total RNA from the high-salt and low-temperature treated (Xu et al., 2014) leaves of BX banana, and then the Matchmaker Gold Yeast OneHybrid Library Screening System Kit (Clontech, Mountain View, CA, United States) was used. Co-transform the bait and pGADT7-Rec prey vector with the cDNA library into Y1HGold (Clontech) cells, and then isolate and place them on SD/-Leu + AbA^200^ medium at 30°C for 3 days. PCR and sequence analysis further verified the selected positive colonies. The identified TF genes are annotated in the banana A genome database.

### Gene Isolation and Sequence Analysis

After BLAST analysis, primers (Supplementary Table 6) and a SMART RACE cDNA Amplification Kit (Clontech) were used to amplify part of the coding sequence (CDS) to obtain a complete CDS. The full-length TF candidate sequence (Supplementary Figure 7) was confirmed and amplified with primers spanning the start and stop codons. These newly isolated TFs were named based on their homologs in Genbank and previously reported TFs in rice.

### Analysis of Dual Luciferase (LUC) Activity

As described by Hellens et al. (2005), the *MaPIP1;1* promoter was cloned into the pGreenII 0800-REN-LUC vector. The primer pair was listed in Supplementary Table 8. The ORFs of the selected interaction genes were transformed into the pGreenII 62Sk vector and then into *Agrobacterium tumefaciens* GV3101 strain. The pGreen-*MaPIP1;1* promoter vector and the *A. tumefaciens* culture containing the expression vector constructed in pGreenII62Sk of each interaction gene were mixed in a 1:8 (v/v) proportion. After injection into the back of tobacco leaves and co-cultivation for 3 days, the luciferase and REN-LUC activities were analyzed using the double LUC reporter gene detection system (Promega, Madison, WI, United States). Three replicates were measured.

### Statistical Analysis Methods

SPSS 10.0 software (SPSS Inc., Chicago, IL, United States) was used to conduct statistical analysis. The least significant test was used for the analysis of variance (ANOVA). The *t*-test was used to test the difference between the methods of analysis of variance. Each sample was composed of three replicates; *p* < 0.05 and *p* < 0.01 were considered to be statistically significant differences.

## Results

### Generation of Banana Plants Overexpressing *MaPIP1;1*

**FIGURE 1:**
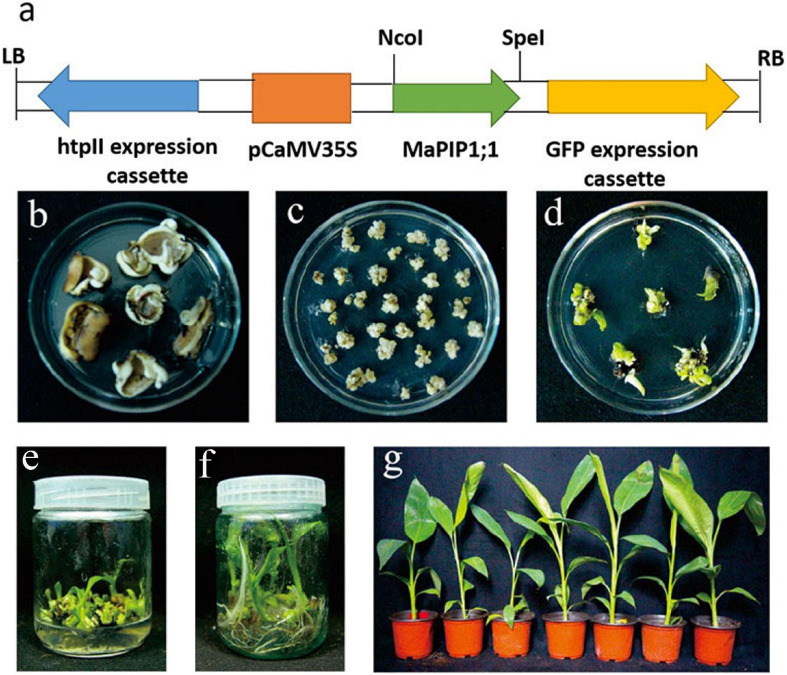
Generation of *MaPIP1;1* overexpressing banana plants. **(a)** Schematic representation of T-DNA region used to generate transgenic plants. **(b)** Slices of the floral apex on differentiation medium. **(c)** Callus regenerated from slices. **(d)** Conversion of callus into shoots on shooting medium. **(e)** Generation of multiple shoots of putative transgenic lines. **(f)** Rooting of different transgenic lines on rooting medium. **(g)** Hardening of rooted transgenic lines.

To study the function of *MaPIP1;1* in bananas, *MaPIP1;1* was transformed into the pCAMBIA1302 expression vector (Figure 1a). The flowers of Gongjiao (*Musa acuminata* L. AA group, cv. Mas) were placed on the differentiation medium to induce the formation of banana callus (Figure 1b).

**FIGURE 2:**
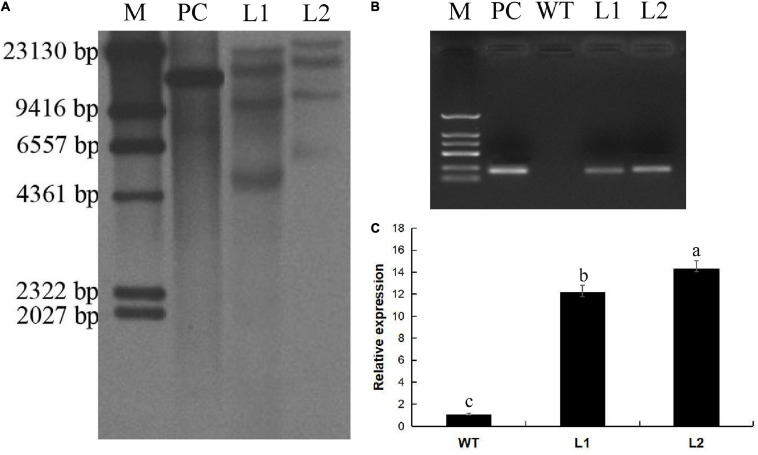
Characterization of the transgenic lines. **(A)** Integration of *MaPIP1;1* transgene in Line 1 (L1) and Line 2 (L2) by Southern blot (35S promoter in the pcambia 1302 vector). **(B)** PCR amplification of the *GFP* gene in transgenic lines. **(C)** qRT-PCR analysis of the expression of *MaPIP1;1* in transgenic lines. Data are means ± SD of *n* = 3 biological replicates. Means denoted by the same letter do not significantly differ at *P* < 0.05 as determined by Duncan’s multiple-range test. M, marker; PC, positive control.

The callus was cut into 2-mm sections and infected with *A. tumefaciens* EHA105 transformed with the *MaPIP1;1*-pCAMBIA1302 expression vector. Murashige and Skoog (MS) medium induced bud growth (Figure 1c). It contained 8.9 μm/L benzylaminopurine (BA), 9.3 μm/L kinetin, 9.1 μm/L zeatin, 9.1 μm/L thiadazolam (TDZ), and 18 mg/L hygromycin (HYG). About 300 pieces of co-culture were used. The explants were placed in a medium (containing 2 mg/L TDZ, 2 mg/L BA, and 18 mg/L HYG) to induce bud differentiation (Figure 1d). When the length of the shoot reached 3 cm (Figure 1e), the explants were transferred to the rooting medium (MS + 4 mg/L BA) to induce root growth. After 30 days of cultivation (Figure 1f), the plants with 8 cm roots were transplanted into coconut peat medium and grown for 100 days (Figure 1g). Finally, 251 hygromycin-resistant transgenic lines were obtained, of which 27 lines were further verified by PCR amplification of the *GFP* gene in the vector. Southern blot analysis and PCR detection verified the L1 and L2 lines. The result of the Southern blot showed that *MaPIP1;1* was successfully transferred to the bananas (Figure 2A). PCR results showed that the GFP gene was only present in the transgenic plants (Figure 2B). These two lines have been carried out vegetative reproduction to expand to the third generation. The expression of *MaPIP1;1* in L1 and L2 transgenic plants was significantly higher than that of wild type (Figure 2C). These results proved that *MaPIP1;1* was successfully overexpressed in bananas.

### Overexpression of *MaPIP1;1* Enhanced Plant Tolerance to Drought Stress

**FIGURE 3:**
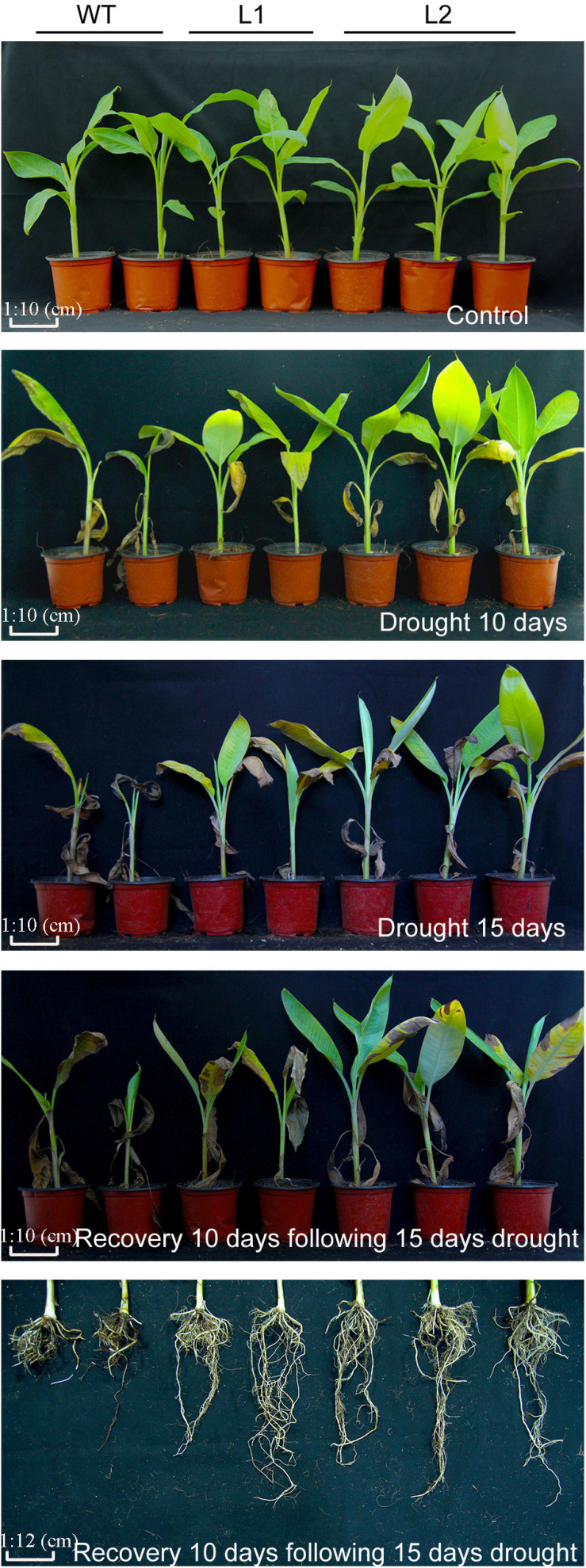
Phenotype differences between WT and transgenic plants under normal, drought, and recovery conditions. Banana seedlings (100-day-old) were subjected to water withholding for 10 and 15 days, and recovery for 10 days following 15 days drought treatment, then the photos were taken.

To detect the performance of *MaPIP1;1* transgenic banana strains under drought stress, the wild type, and *MaPIP1;1-*high-expressing strains grown for 100 days were subjected to drought stress. After 10 or 15 days of water shortage, the leaves of most WT plants were curled and chlorotic, while most of the leaves of the *MaPIP1;1* transgenic lines remained green. After 15 days of drought treatment and re-watering for 10 days, the transgenic plants showed better growth vigor, with more green leaves and less damage to the root system (Figure 3). The above results indicate that *MaPIP1;1* improves the drought tolerance of transgenic plants.

**FIGURE 4:**
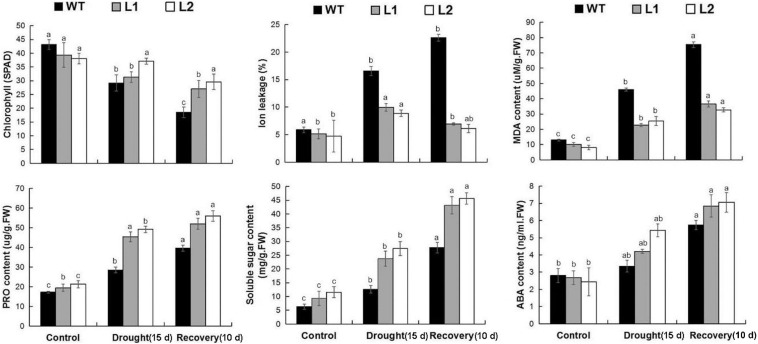
Comparisons of physiological indices between WT and transgenic plants under normal, drought, and recovery conditions. Control means banana seedlings (100-day-old) grow normally. Drought means banana seedlings (100-day-old) were subjected to water withholding for 15 days, and recovery means banana seedlings (100-day-old) growth recovery for 10 days following 15 days drought treatment. Banana seedlings (100-day-old) were subjected to water withholding for 15 days and recovery for 10 days following 15 days drought treatment. The leaves were then sampled to perform physiological analyses. Data are means ± SD of *n* = 3 biological replicates. Means denoted by the same letter are not significantly different at *P* < 0.05 as determined by Duncan’s multiple-range test.

Next, we examined the MDA level in the plant to study the physiological mechanism that *MaPIP1;1* confers in improving the drought tolerance of transgenic bananas. The MDA content in plants is a sign of damage mediated by reactive oxygen species (ROS) (Moore and Roberts, 1998). IL is an indicator of membrane damage. In addition, we measured the content of soluble sugar and proline, which are representative substances that can protect cells under stress conditions (El-Esawi et al., 2019; Xu et al., 2020b), and the contents of ABA (Supplementary Table 9). ABA is a hormone that responds to plant stress. Under normal growth conditions, compared with WT, transgenic plants had lower MDA and IL content and higher soluble sugar content. After drought stress treatment and recovery, the levels of MDA and IL in the transgenic plants were still lower. The contents of proline, chlorophyll, soluble sugar, and ABA in the transgenic plants were higher (Figure 4). Therefore, the overexpression of *MaPIP1;1* led to a reduction in membrane damage and the osmotic balance of plant cells and ABA levels under drought stress.

### Overexpression of *MaPIP1;1* Increases Plant Tolerance to Salt Stress

**FIGURE 5:**
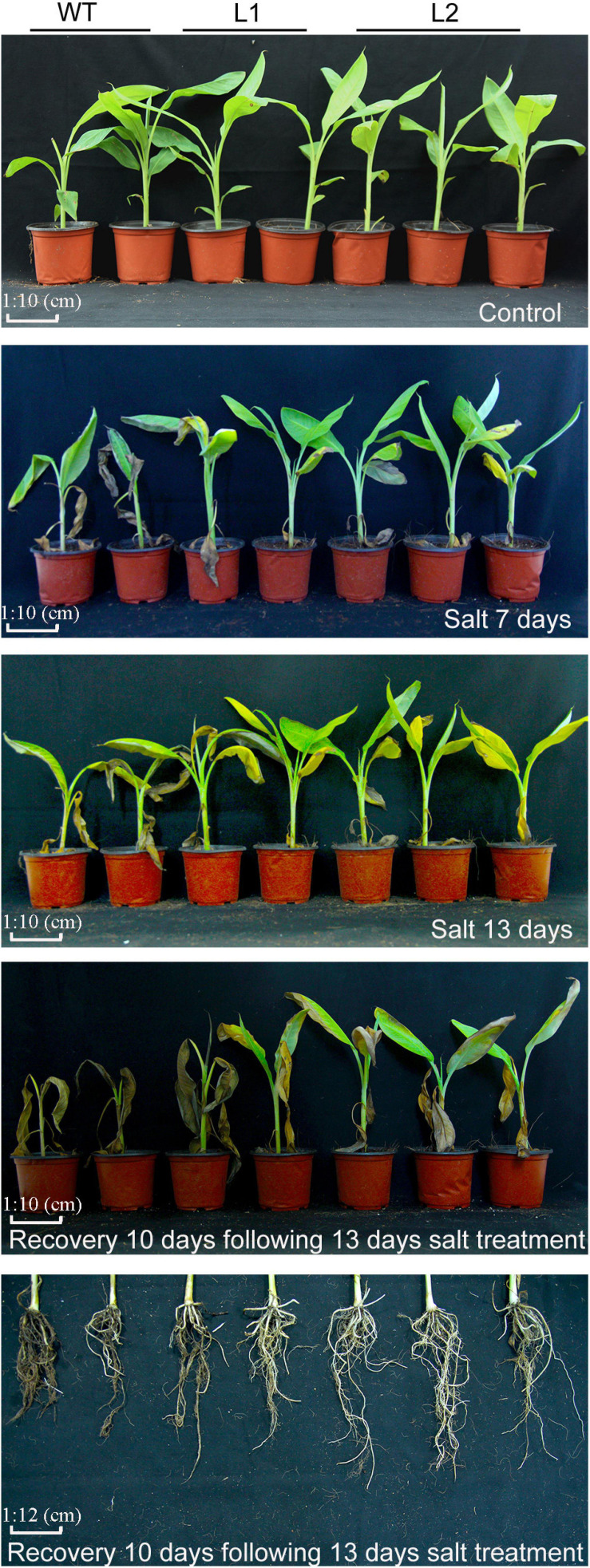
Phenotype differences between WT and transgenic plants under normal, salt, and recovery conditions. Banana seedlings (100-day-old) were subjected to salt treatment (250 mM NaCl) for 7 and 13 days, and recovery for 10 days following 13 days salt treatment, then the photos were taken.

To further examine whether the transgenic plants can improve salt tolerance, after 7 and 13 days of salt treatment, the transgenic lines showed better vigor, while their leaves were less wilted and yellowed. After 13 days of high-salt stress treatment and 10 days of recovery, the transgenic lines had more green leaves and less root damage than WT (Figure 5), indicating that *MaPIP1;1* overexpression can improve the salt stress tolerance of plants.

**FIGURE 6:**
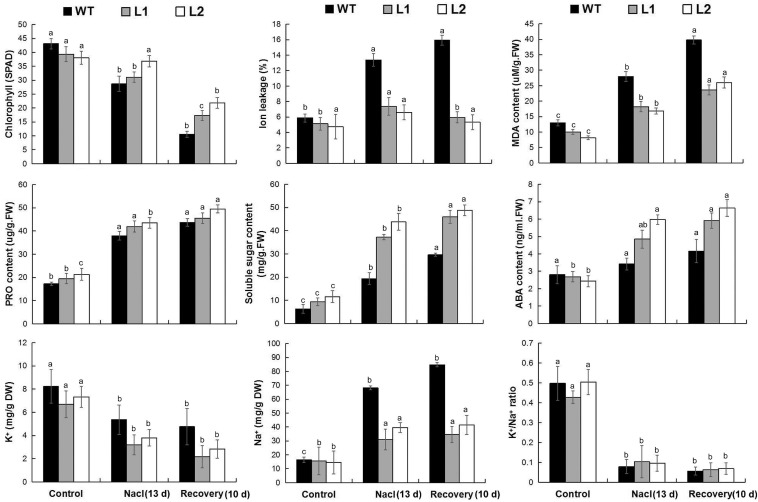
Comparisons of physiological indices between WT and transgenic plants under normal, salt, and recovery conditions. Control means banana seedlings (100-day-old) grow normally. NaCl means banana seedlings (100-day-old) were subjected to salt treatment (250 mM NaCl) for 13 days, and recovery means banana seedlings (100-day-old) growth recovery for 10 days following 13-day salt treatment. Data are means ± SD of *n* = 3 biological replicates. Means denoted by the same letter are not significantly different at *P* < 0.05 as determined by Duncan’s multiple-range test.

In terms of physiological response, the transgenic plants had lower IL and MDA contents, higher chlorophyll, proline, soluble sugar, and ABA contents under salt stress and recovery (Supplementary Table 10). In addition, under salt stress treatment and recovery conditions, *MaPIP1;1* overexpression reduced the contents of Na^+^ and K^+^ in the cells, while the K^+^/Na^+^ ratio increased during the recovery process (Figure 6). These results showed that *MaPIP1;1* reduces the membrane damage of transgenic bananas, improved ABA levels, and affected the contents of Na^+^ and K^+^ at the cellular level under salt stress.

### Overexpression of *MaPIP1;1* Improves Cold Stress Tolerance

**FIGURE 7:**
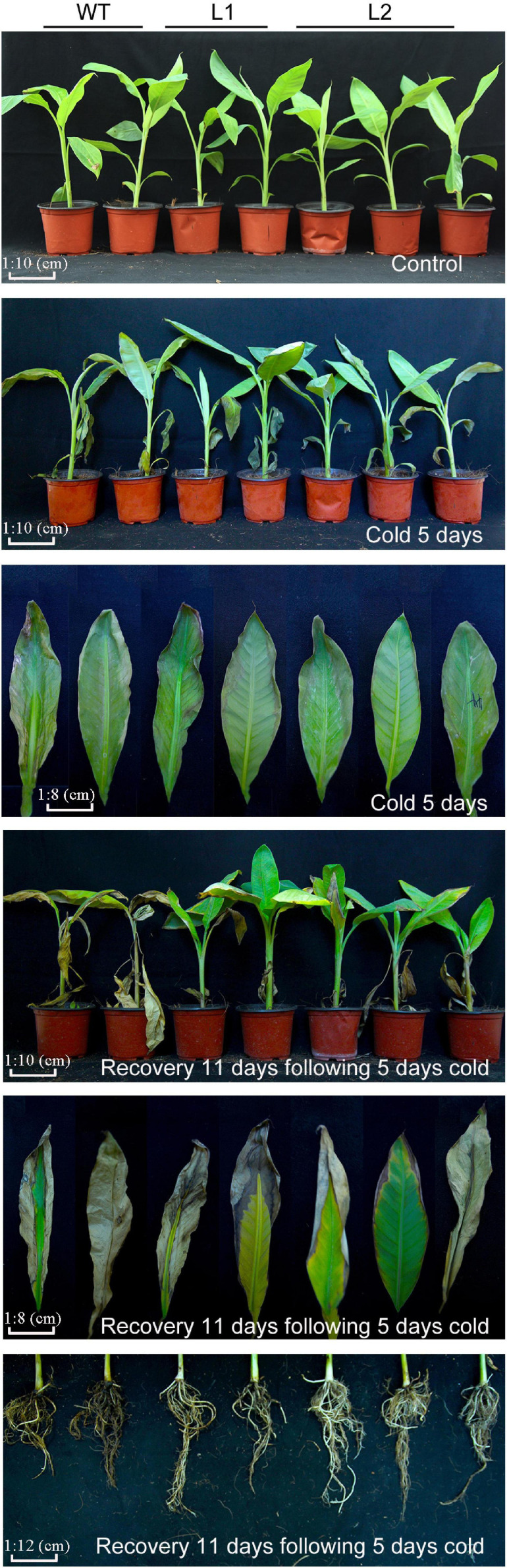
Comparison of phenotypic differences between wild-type (WT) and transgenic lines under normal, cold, and recovery conditions. Banana seedlings (100-day-old) were subjected to cold treatment (8°C) for 5 days and recovery for 11 days after 5-day cold treatment. Then the photos were taken.

To test the cold resistance of the transgenic plants, bananas were treated with low temperatures. After 5 days of low temperature (8°C) treatment, WT leaves are more curled and withered than that of transgenic plants. After 11 days of recovery, the WT plants showed severe yellowing of leaves, while the transgenic lines showed greater vitality. In addition, under low temperature and recovery conditions (Figure 7), the transgenic line showed less root damage than the wild type. These results showed that *MaPIP1;1* improves the cold tolerance of transgenic bananas.

**FIGURE 8:**
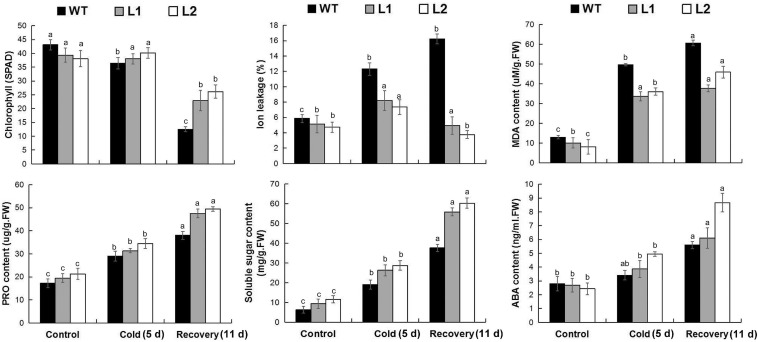
Comparisons of physiological indices between WT and transgenic plants under normal, cold, and recovery conditions. Control means banana seedlings (100-day-old) grow normally. Cold means banana seedlings (100-day-old) were subjected to cold treatment (8°C) for 5 days, and recovery means banana seedlings (100-day-old) growth recovery for 11 days following 5-day cold treatment. The leaves were then sampled to perform physiological analyses. Data are means ± SD of *n* = 3 biological replicates. Means denoted by the same letter are not significantly different at *P* < 0.05 as determined by Duncan’s multiple-range test.

We further tested the physiological mechanism of *MaPIP1;1* improving plant cold tolerance under low-temperature conditions. Under normal growth conditions, compared with the control, the soluble sugar content of the transgenic bananas is higher, while the levels of MDA and IL are lower. After 5 days of low-temperature treatment and 11 days of the recovery period, the content of MDA and IL are lower in the transgenic plant. In the transgenic banana, the contents of chlorophyll, proline, soluble sugar, and ABA were higher than that in the wild type (Figure 8 and Supplementary Table 11). Therefore, *MaPIP1;1* can reduce the ABA content and enhance osmotic balance in plant cells in transgenic plants.

### Overexpression of *MaPIP1;1* Affects the Expression of ABA Biosynthetic and Responsive Genes

**FIGURE 9:**
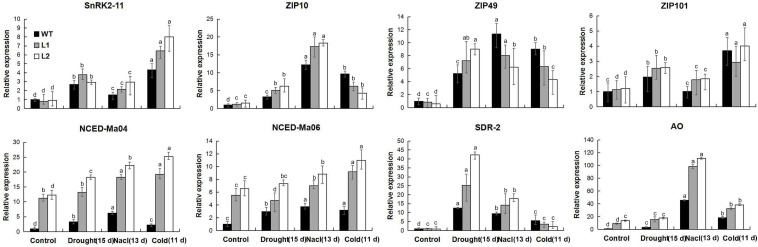
Expression levels of ABA biosynthetic and responsive genes in WT and transgenic lines under normal, drought, cold, and salt conditions. Control means banana seedlings (100-day-old) grow normally. Drought, NaCl, and Cold means, respectively, banana seedlings (100-day-old) were subjected to water withholding for 15 days, salt (250 mM NaCl) for 13 days, and cold (8°C) for 5 days. Data are means ± SD of *n* = 3 biological replicates. Means denoted by the same letter are not significantly different at *P* < 0.05 as determined by Duncan’s multiple-range test.

Abscisic acid plays a vital role in improving the drought, salt, and low-temperature resistance of plants. Under different stress treatments and recovery conditions, physiological research has found that *MaPIP1;1* can increase the level of ABA in plants. Therefore, we detected the expression of ABA biosynthetic and responsive genes in WT and transgenic plants by qRT-PCR (Supplementary Table 12). The ABA biosynthesis genes (*MaNCED-Ma04*, *MaNCED-Ma06*, and *MaAO*) and response genes (*MaSnRK2-11* and *MabZIP101*) in the overexpression plant showed higher expression under the drought, high salt, and low-temperature treatment (Figure 9). Other tested genes showed stress-specific overexpression induced by *MaPIP1;1*. Other tested genes showed that the expression of *MaSDR2* is induced under drought and NaCl treatment, high expression of *MabZIP10* under NaCl stress, and high expression of *MabZIP49* in transgenic lines under drought stress. These results further proved that the overexpression of *MaPIP1;1* activated ABA biosynthesis and signal transduction.

### Screening and Identifying the Transcription Factors Binding to *MaPIP1; 1* Promoter in Yeast

To further analyze the mechanism of *MaPIP1;1* in improving plant resistance under high-salt and low-temperature stress, a pre-built *MaPIP1;1* promoter M-P2 bait vector (Xu et al., 2020a) in high-salt and low-temperature-treated banana cDNA one-hybrid libraries was used to screen interacting genes (Supplementary Figure 13).

**FIGURE 10:**
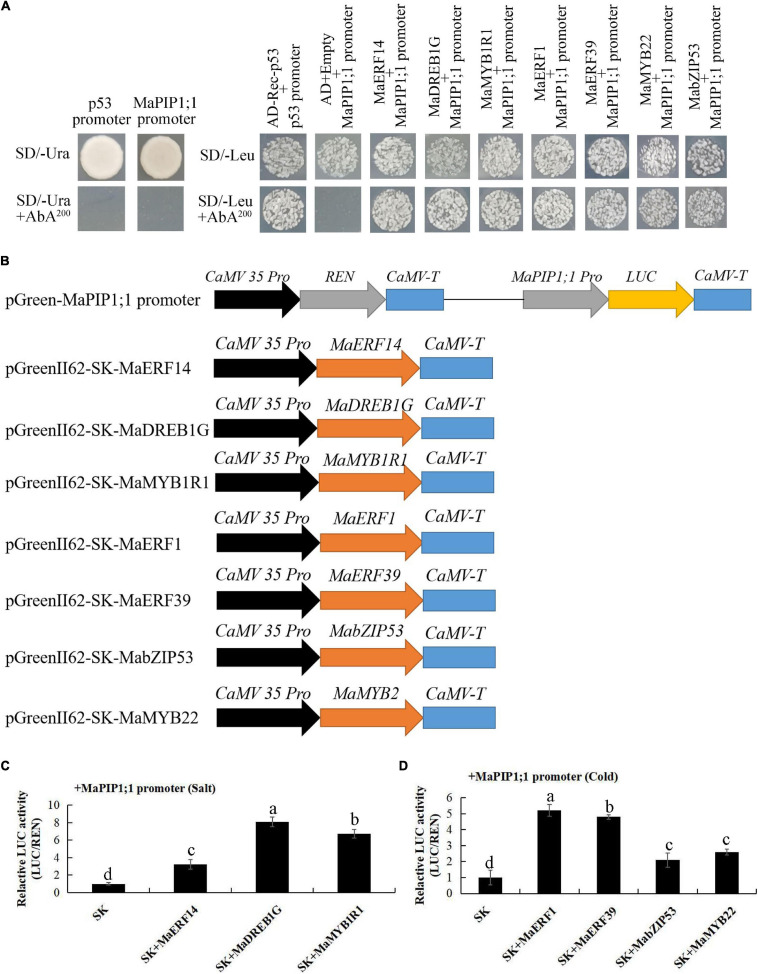
Activation of the *MaPIP1;1* promoter by the transcription factors. **(A)** Physical interactions between MaERF14, MaDREB1G, MaMYB1R1, MaERF1/39, MabZIP53, MaMYB22, and the *MaPIP1;1* promoter according to yeast one-hybrid analysis. The autoactivation of the promoters was determined on SD/-Ura + AbA media, while the interaction between TFs and the promoter was tested on SD/-Leu + AbA media. **(B)** Schematics of transient expression vectors. **(C,D)** LUC activity resulting from the transient co-expression of the *MaPIP1;1* promoter and the transcription factors in *Agrobacterium tumefaciens* strains. There were three replicates. The data are presented as the means ± SDs. Means denoted by the same letter are not significantly different at *P* < 0.05 as determined by Duncan’s multiple-range test.

The colonies were further flat coating on plates containing 3-amino-1,2,4-triazole (3AT). Under high salt stress, 23 colonies were screened and sequenced (Supplementary Table 14). Among them, there were one MaERF, one MaDREB, and one MaMYB. In subsequent verification using individual Y1H assays. In separate one-to-one Y1H analysis in subsequent verification, the yeast clones harboring pAbAi-MaPIP1;1 + pGADT7-MaERF14, pAbAi-MaPIP1;1 + pGADT7-MaDREB1G, and pAbAi-MaPIP1;1 + pGADT7-MaMYB1R1 grew normally on SD/-Leu + AbA^200^ selective medium. The result was the same with the library screening (Figure 10A). Under the cold treatment, a total of 25 interacting clones were screened, and the sequencing results are shown in Supplementary Table 15: one MaMYB, two MaERFs, and one MabZIP. In one-to-one Y1H analysis, pAbAi-MaPIP1;1 was with the pGADT7-MaMYB22/MaERF1/MaERF39/MabZIP53, normal growth on SD/-Leu + AbA^200^ selective medium, which was consistent with the results of hybrid library screening (Figure 10A).

### Activation of the *MaPIP1;1* Promoter by MaMYB1R1, MaERF14/1/39, MaDREB1G, MabZIP53, and MaMYB22

In the early yeast one-hybrid screening, we obtained transcription factors that interacted under high-salt and low-temperature conditions: two MaMYBs, one MabZIP, one MaDREB1G, and three MaERFs bound to *MaPIP1;1 in vitro*.

The *MaPIP1;1* promoter M-P2 fragment was cloned into a reporter pGreen II 0800 vector termed pMaPIP1;1:LUC, and the full-length ORF for each transcription factor was cloned into the effector vector pGreenII 62Sk (Figure 10B).

Each gene and the *MaPIP1;1* promoter were co-transformed into GV3101 *Agrobacterium* and injected into tobacco leaves for expression. The results showed that all nine transcription factors screened combined with the *MaPIP1;1* promoter to increase LUC activity (Figures 10C,D).

### Expression of the *MaPIP1;1* Promoter-Binding Transcription Factors Under Salt and Cold Stress

**FIGURE 11:**
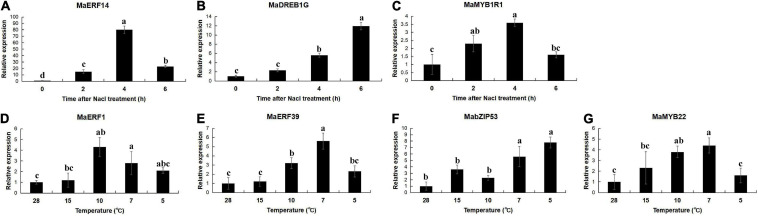
Expression analysis of the binding transcription factors under the salt and cold stresses in the banana. Data are means ± SD of *n* = 3 biological replicates. Means denoted by the same letter are not significantly different at *P* < 0.05 as determined by Duncan’s multiple-range test.

The expression of the selected interaction genes *MaERFs*, *MaDREB*, *MaMYBs*, and *MabZIP* had been analyzed in the five-leaf, and one-heart banana leaves treated with high salt and low temperature. As can be seen from the results, under salt stress, the expressions of *MaERF14*, *MaDREB1G*, and *MaMYB1R1* were significantly induced. The expressions of *MaERF1/39*, *MaMYB22*, *MabZIP53* were induced under the cold stress (Figure 11). This is consistent with the functional description of these genes under adverse conditions.

## Discussion

Many abiotic stresses will affect the normal growth and development of crops, which will affect their yield and quality. Bananas are also susceptible to these abiotic stresses (van Asten et al., 2011; Sreedharan et al., 2013). Therefore, identifying genes that can improve the ability of plants to resist abiotic stresses and analyzing their gene functions and mechanisms are important ways to carry out more effective molecular breeding (Xu et al., 2020a). Molecular breeding is an important way to obtain resistant banana varieties, and genetic modification is an important means. Due to the long and cumbersome process of banana transgenics, there are few reports on banana transgenics. In addition, the research on the mechanism of transgenics to improve banana resistance is still insufficient. There are a few studies on genetically modified bananas in existing reports. *Mu-saPIP1;2* can improve the drought, salt, and cold tolerance of transgenic bananas (Sreedharan et al., 2013). Overexpression of *MusaPIP2;6* gene in banana can improve the salt tolerance of the plant (Sreedharan et al., 2015). In *MusaVND1* transgenic bananas, genes related to the biosynthetic pathway of lignin and cellulose were significantly induced to express (Negi et al., 2015). In the *AhcAPX* transgenic banana, the salt tolerance and drought resistance of the plant is improved by reducing the damage caused by ROS (Shekhar et al., 2019). In a previous study, we also obtained resistant transgenic banana plants. The *MaPIP2-7* transgenic banana has improved its drought, salt, and cold tolerance (Xu et al., 2020c). When transformed into *MaSIP2-1*, it can improve the drought and cold tolerance of genetically modified bananas (Xu et al., 2020b).

Water regulation and transport play a very important role in improving the exposure of crops to abiotic stresses such as salt, drought, and cold (Zhang et al., 2019). The AQPs of the plant can promote the transport of water in cell membranes and improve the ability of plants to resist abiotic stress. Early studies have shown that the PIPs genes in the AQP family of many plants play an active role in promoting water transport through the plasma membrane (Corey et al., 2016). At the same time, it participates in many abiotic stress responses (Maurel, 1997). Our previous research showed that AQPs could reduce the impact of external abiotic stress, the *MaPIP1;1* overexpression plants have stronger resistance under drought and high salt stresses (Xu et al., 2014). This study proved that *MaPIP1;1* could improve the drought resistance, salt tolerance, and low-temperature resistance of genetically modified bananas. In addition, the *MaPIP1;1* transgenic plants showed better growth vigor under the restored conditions (Figures 3, 5, 7). Therefore, *MaPIP1;1* can improve the stress resistance of plants under both stress and recovery conditions. In contrast, some AQPs exhibit negative functions in overexpressing plants. For example, under drought stress, *AtPIP1;b* leads to rapid wilting of overexpressed tobacco (Aharon et al., 2003). Plants overexpressing the *Arabidopsis* genes *AtPIP1;4* and *AtPIP2;5* can cause dehydration under drought stress (Jang et al., 2007). Therefore, different members of the AQP family show obvious functional differences.

Drought, salt, and cold stresses can disrupt the osmotic balance of plants. To cope with these stresses, there will be some accumulation of related substances in plants (Miao et al., 2020); proline and soluble sugar accumulation is extensive. The accumulation of these substances protects the cells by reducing the osmotic pressure of the cells in the plant (El-Esawi et al., 2019). In this research, compared with the wild type, *MaPIP1;1* transgenic bananas had higher proline and soluble sugar contents, indicating that it can improve the adaptability of transgenic plants under drought, salt, and cold stresses and recovery conditions. This is consistent with the situation where we previously expressed the AQP *MaPIP2-7* and *MaSIP2-1* in bananas (Xu et al., 2020a,b). Abiotic stress usually oxidizes DNA, lipids, and proteins, which causes the rapid accumulation of ROS (Mittler et al., 2004). MDA and IL are two important indicators for evaluating ROS-mediated cell damage under stress conditions (Sreedharan et al., 2013). The result showed that overexpression of *MaPIP1;1* reduced the accumulation of MDA and IL, indicating lipid peroxidation in the plants overexpressing *MaPIP1;1* under drought, salt, and cold stresses, and the membrane is less damaged (Figures 4, 6, 8). Consistent with our results, related reports showed that rice plants overexpressing *OsPIP2;7* had a lower IL content under low-temperature stress, and overexpression of *TaAQP7* reduced the content of MDA and IL under drought conditions (Zhou et al., 2012). Therefore, *MaPIP1;1* improves the stress resistance of overexpression plants by reducing membrane damage and oxidation under stress conditions. Na^+^ harms cell metabolism and certain proteins. A high Na^+^ content will also reduce the photosynthesis of plants and cause oxidative damage (Mahajan and Tuteja, 2005). In this study, we detected the accumulation of K^+^ and Na^+^ in *MaPIP1;1* overexpression plants and WT plants, respectively. The figure shows that under salt stress, the K^+^ and Na^+^ in the cells of *MaPIP1;1* transgenic plants decreased, while the ratio of K^+^/Na^+^ increased. It is generally believed that a high K^+^/Na^+^ ratio in cells can improve the salt tolerance of plants (Ruiz-Lozano et al., 2012). *TaNIP* or *TaAQP8* will affect the distribution of Na^+^ and K^+^ contents in the transgenic plants under salt stress and increase their K^+^/Na^+^ ratio (Gao et al., 2010; Hu et al., 2012). Therefore, *MaPIP1;1* reduces the Na^+^ and K^+^ content of transgenic plants and increases the K^+^/Na^+^ ratio to improve the salt tolerance of plants.

Hormones are also involved in the process of plants responding to abiotic stress. The biosynthesis and signal transduction of ABA play a positive role in improving drought resistance of plants, high-salt, and low-temperature stress (Ben-Ari, 2012). Physiological research results showed that the expression of *MaPIP1;1* increased ABA levels in plants under stress treatment and recovery. Therefore, we tested the expression of genes related to ABA biosynthesis in control and transgenic plants under normal and stress conditions. Compared with the wild type, ABA biosynthesis genes (*MaNCED-Ma04*, *MaNCED-Ma06*, and *MaAO*) and response genes (*MabZIP49* and *MaSnRK2-11*) showed higher expression levels in the transgenic plants under drought, salt, and low-temperature conditions.

We further analyzed the molecular mechanism of *MaPIP1;1* in improving the resistance of genetically modified bananas. In a previous study, we screened out the transcription factor MaMADS3 that interacted with the yeast one-hybrid under drought stress (Xu et al., 2020a). The mechanism of the same gene response to stress under different adversity conditions might be regulated differently. In this study, we screened transcription factors that interacted with *MaPIP1;1* promoter under high-salt and low-temperature stresses. Under high-salt stress, we screened transcription factors MaERF14, MaDREB1G, and MaMYB1R1 to interact with its promoter, and under low-temperature stress conditions, we screened MaERF1/39, MabZIP53, and MaMYB22. At the same time, we found that under different stress conditions of high salt and low temperature, although the results included the ERF and MYB families, their family members were also different.

Numerous researches have indicated that transcription factors play active roles in improving plant resistance to various abiotic stresses. The ethylene-responsive factor (ERF) family encodes transcription regulators with multiple functions involved in plant development and physiological processes (Nakano et al., 2006). The apple ERF transcription factor MdERF38 can promote anthocyanin biosynthesis induced by drought stress (An et al., 2020). Li reported that the tomato ERF gene *SlERF84* could improve the adaptability of transgenic *Arabidopsis* to drought and high salt (Li et al., 2018). Another binding transcription factor is dehydration-responsive element-binding (DREB), which belongs to the AP2/ERF family. It is also widely involved in the process of improving plant abiotic stress ability. Overexpression of *ScDREB10* in the desert moss *Syntrichia caninervis* significantly improved the tolerance of transgenic *Arabidopsis* thaliana to osmotic and salt stresses in the seedling stage (Li X. S. et al., 2019). The v-myb avian myeloblastosis viral oncogene homolog transcription factor (MYB) participates in the growth and development of plants and responds to abiotic stress. *Arabidopsis AtMYB44* and *AtMYB2* can increase the resistance of transgenic plants (Jung et al., 2008). The survival rate of *GmMYB76* transgenic soybean after cold stress was much higher than that of the WT, showing that it can improve the ability of plants to resist low temperature (Liao et al., 2008). The basic region/leucine zipper (bZIP) family members can improve the drought resistance of transgenic plants (Tu et al., 2016a,b; Ma et al., 2018). The research of Tu et al. (2018) showed that the grape bZIP gene *VlbZIP30* could improve the drought resistance of transgenic *Arabidopsis* and grape seedlings.

In general, the interaction between transcription factors and the gene promoters depends on specific binding sequences, also named *cis*-acting elements. Studies have shown that ERF binds to the ethylene response element (ERE) with the core sequence of AGCCGCC (also known as the GCC-box), thereby conferring resistance to biological stress (Shinozaki and Yamaguchi-Shinozaki, 2000; Franco-Zorrilla et al., 2014). Sun’s research shows that grape VaERF092 enhanced the tolerance to low-temperature stress by combining with the GCC-box in the *VaWRKY33* promoter (Sun et al., 2019). DREB can identify dehydration sensitive or the C repeat elements (DRE/CRT) A/GCCGAC core sequence, thus responding to abiotic stresses. Relevant studies have shown that MYB can improve the stress resistance of plants by combining with TAACCA elements in the promoters of downstream genes. MYB15 binds and interacts with the binding site of the *ICE1* gene promoter in *Arabidopsis* (Li J. L. et al., 2019). The studies have shown that certain bZIP proteins regulate their expression by binding to the ACGTG element in the target genes promoters (Ezer et al., 2017; Ma et al., 2018). Grape VlbZIP30 initiates gene expression by combining G-box *cis*-elements in the *VvPRXN1* and *VvNAC17* promoters (Tu et al., 2020).

In this study, we screened transcription factors that bind to *MaPIP1;1* under salt and cold stresses. In the promoter region of *MaPIP1;1*, the binding element sequences AGCCGCC, ACCGAC, TAACCA, and ACGTG are supposed to be recognized by ERF, DREB, MYB, and bZIP, respectively (Supplementary Figure 13). This result also provides evidence for the interaction between promoters and transcription factors. This is also the first report that AQP genes combine with these transcription factors to improve plant stress resistance. In summary, identifying these TFs lays a foundation for further elucidating the molecular regulation mechanism of *MaPIP1;1* transgenic bananas under abiotic stress.

**FIGURE 12:**
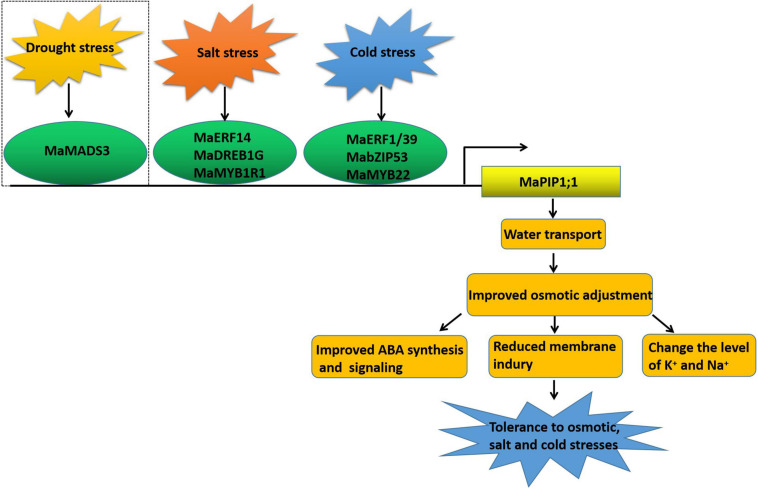
*MaPIP1;1* combines with transcription factors under different abiotic conditions to improve the drought, salt, and low-temperature tolerance of transgenic bananas. The dotted line indicates the previous work.

In conclusion, here we have shown that *MaPIP1;1* improves the drought resistance, salt tolerance, and low-temperature tolerance of transgenic bananas by improving banana osmotic regulation and reducing membrane damage. In addition, the activation of *MaPIP1;1* by salt stress plays a certain role in reducing the contents of Na^+^ and K^+^ in plants and increasing the ratio of K^+^/Na^+^, which is beneficial for improving the salt tolerance of plants. In addition, the transcription factors MaERFs, MaDREB, MaMYBs, and MabZIP interact with the *MaPIP1;1* promoter identified under high salt and low-temperature stress further reveals the mechanism of *MaPIP1;1* improving plant resistance (Figure 12). This research provides a theoretical basis for mining resistance-related genes and understanding their mechanism of action and has laid a good foundation for resistance breeding.

## Data Availability Statement

The original contributions presented in the study are included in the article/Supplementary Material, further inquiries can be directed to the corresponding authors.

## Author Contributions

YX, BX, and ZJ were conceived and designed the experiments. YX and SS were performed the experiments, analyzed the data, and wrote the article. CJ and WH were prepared the figures and tables. YX and JL were contributed reagents, materials, and analysis tools and reviewed drafts of the article. All authors contributed to the article and approved the submitted version.

## Conflict of Interest

The authors declare that the research was conducted in the absence of any commercial or financial relationships that could be construed as a potential conflict of interest.

## Publisher’s Note

All claims expressed in this article are solely those of the authors and do not necessarily represent those of their affiliated organizations, or those of the publisher, the editors and the reviewers. Any product that may be evaluated in this article, or claim that may be made by its manufacturer, is not guaranteed or endorsed by the publisher.
