# Erratum: Overexpression of a Banana Aquaporin Gene *MaPIP1;1* Enhances Tolerance to Multiple Abiotic Stresses in Transgenic Banana and Analysis of Its Interacting Transcription Factors

**DOI:** 10.3389/fpls.2021.780544

**Published:** 2021-10-06

**Authors:** Frontiers Production Office

**Affiliations:** Frontiers Media SA, Lausanne, Switzerland

**Keywords:** *MaPIP1*, tolerance, banana, interaction, transcription factor

Due to a production error, there were errors in the affiliations in the originally published article. Instead of

“Yi Xu^1,2,3^, Juhua Liu^4^, Caihong Jia^4^, Wei Hu^4^, Shun Song^1,2,3*^, Biyu Xu^4*^ and Zhiqiang Jin^4*^

^1^Key Laboratory of Genetic Improvement of Bananas, Haikou Experimental Station, Chinese Academy of Tropical Agricultural Sciences, Haikou, China

^2^Sanya Research Institute of Chinese Academy of Tropical Agricultural Sciences, Sanya, China

^3^Hainan Key Laboratory for Biosafety Monitoring and Molecular Breeding in Off-Season Reproduction Regions, Sanya, China

^4^Key Laboratory of Biology and Genetic Resources of Tropical Crops, Institute of Tropical Bioscience and Biotechnology, Chinese Academy of Tropical Agricultural Sciences, Haikou, China”,

the affiliations should have been presented as

“Yi Xu^1,3,4^, Juhua Liu^2^, Caihong Jia^2^, Wei Hu^2^, Shun Song^1,3,4*^, Biyu Xu^2*^ and Zhiqiang Jin^2*^

^1^Key Laboratory of Genetic Improvement of Bananas, Haikou Experimental Station, Chinese Academy of Tropical Agricultural Sciences, Haikou, China

^2^Key Laboratory of Biology and Genetic Resources of Tropical Crops, Institute of Tropical Bioscience and Biotechnology, Chinese Academy of Tropical Agricultural Sciences, Haikou, China

^3^Sanya Research Institute of Chinese Academy of Tropical Agricultural Sciences, Sanya, China

^4^Hainan Key Laboratory for Biosafety Monitoring and Molecular Breeding in Off-Season Reproduction Regions, Sanya, China”.

The publisher apologizes for this mistake. The original article has been updated.

