# Supplementary material for: Overexpression of a Banana Aquaporin Gene MaPIP1;1 Enhances Tolerance to Multiple Abiotic Stresses in Transgenic Banana and Analysis of Its Interacting Transcription Factors
Source: Front Plant Sci. 2021 Aug 25;12:699230. doi: 10.3389/fpls.2021.699230 (PMC8424054; doi:10.3389/fpls.2021.699230)
Supplement: Supplementary file 4 [file Table_4.DOCX]

**Fig. S4**  Sequences of MaSnRK2-11, MaZIP10, MaZIP49, MaZIP101 and MaNCED-04，MaNCED-06，MaSDR-2，MaAO

**1.MaSnRK2-11（1020 bp）**

ATGGAGGAGAGGTACGAGCTGCTGAAGGAGCTGGGAGCCGGGAACTTTGGGGTGGCGAGGCTGGTGAAGGACAAGAAGACGGGCGAGCTCGTTGCAGTCAAGTACATCGAAAGGGGGAAGAAGATCGATGAGCATGTGCTGAGAGAAATAATTAACCACAAGTCCTTGAGGCATCCTAATATCGTCCGGTTCAAGGAGGTAGTGCTGACACCAACACATCTAGCCATAGTCATGGAATACGCTGCTGGTGGTGAACTCTTTGAGAGGATATGCAGTGCCGGTCGGTTTAGTGAAGATGAGGCAAGGTATTTCTTTCAGCAGCTGATATCGGGAGTCAGTTATTGTCATTCTATGGAAATCTGTCACCGTGATCTTAAACTCGAGAATACACTCTTGGACGGAAGTCCAACACCGCGTGTTAAAATATGTGACTTTGGGTACTCAAAGTCTGCTTTGTTGCATTCACAACCCAAGTCAACAGTGGGTACTCCAGCCTACATCGCACCGGAAGTACTATCAAGAAAAGAATACGATGGCAAGATTGCAGATGTTTGGTCTTGTGGTGTCACATTGTACGTGATGTTGGTTGGCTCATACCCATTCGAGGATCCCGAAGATCCCAGAAACTTCCGGAAGACTATAAGTAGAATACTAAATGTTCAGTACTCCGTCCCCGATTATATTCGTATATCTTCAGAGTGCCGGCAACTTCTATCACAAATTTTCGTCGCCAACCCATTGAAGAGGATTACAATTCCGGAGATAAAACAGCATCCTTGGTTTCTAAAGAACCTTCCCAAAGAAATCATAAATGGTGAGAAGACAAACTTCGAAGGCAACGATGATCAGCCCTCCCAGAGCGTCGAAGACATCATGCGCATCATTGAAGAGGCAAAGAAGCCGGCAGAATACCCCAAAGTGGCCAACAATCCAGTTTCGGAGTTGGTGGAAGTTGATGAAGTCGATCCCGACGATGAGACCGAAGTCGAAAGCAGTGGGGATTTCCTAGAATCCATTTGA

**2.MaZIP10（1212 bp）**

ATGAATTCCAAGAGTTTGGGAGTAGGAAGAGAGGATGGACAGGCGCAGCCACCACCGCCGCCGACGCTGCTGTCGCGGCAGGGTTCGATTTACTCGCTGACGTTCGACGAGTTCCAGAGCACGCGCGGGGGGCTTGGCAAGGACTTCGGGTCGATGAACATGGACGAGTTCCTTAAGAACATATGGACCGCGGAGGAGAGCTACGCCATGGTCGCCGCCCTCGGCGACGGCAGCGGTGGCTTTGGTGCCGGGGCCGGCCTCCAGCGGCAGGCCTCGCTCACCTTGCCCAGAACCCTTAGCCAGAAGACTGTCGACCAGGTCTGGCGGGGCCTCGTCGAACCCTCCTCCTCCGGCCAAGGGGTGGCCGCCAGCTGCGGCGGCACGGACTTCCCGCGGCAACCCACCCTCGGGGAGATTACCCTTGAGGAGTTCTTGGTGCGGGCCGGAGTGGTGCGGGAGGATATGGCTCCGTCGCCGAGACCACCGACGCCGATTGGCAATAAAAGCAACAACACCAATATCTACTATGGAGATTTGCCGGTCGTGAACACTTCGGCTGGGCTGGAACTCAAGTTCAATCAGGCACCGGGTCGAAGCAATGGAAATATGGCAAACGTTCCAATTGCTCACGGTTCAGCTGCTAATTTGGGGGTGACATCCACTGTTGCGAGACCTTTTGCGCCTCCAGTTCCCCTGGGAGATAGCATGGGCTTGGTGAGCCCGCAGGGCATGAGAGGCGGAGAACTTGGCGGCTTTGGCCACGTCGGGATGAACAACAGATTGATGACGGGGATGGTTGGTCTGAGCACAGCTGGAGTCATGGGAGCGTCAGGGTCGCCAAAAAACCATCTGTCTTCAGATGAAATTGTTAAAGGCAACGGGGATCTATCTTCTTTATCACCTGTTCCATATGTGTTTAATGGTGGGCCGAGGGAAAGGAAGCGTAATAGGAGTCTGGATAAAGTTGTGGAAAGGAGGCAGAGGAGGATGATCAAGAACAGGGAGTCAGCTGCTAGATCACGTGCCCGGAAGCAGGCTTATACTGTGGAGTTGGAAGCTGAAGTGGCAAAACTCAAAGAGCTAAACCAAGAATTGCAGAAAAAACAGGTGGAAATGATGGAGATGAAGAAGAATCAGGTCCTCCAGGTGATCAAGCGGCAGCATGGACAAAAGAAACAACGGTTGAGGAGGACACGAACGGGTCCATGGTAA

**3.MaZIP49（705 bp）**

ATGGCTGATCATGGCGGCGGCCGGGATCAGCAGTTGCAGCCTCTGACCGACGCCGAACACGGTTCCTTCCACGGTCTCATGCTCAACGAGGTCCAGAGCCGCCTGGGGGGGCCCCTGCACGGCCTGAGCCTCGGCGACCTCCTCGAGCATGCCCCTCTCGTCGCCGACGGGCTCCGTTGCAGTTGGAGCGGCGGCGTGCCGCGTGTCCTGAGCAAGAAGACCATTGTCGAGGCGTGGAGGGACATCCAGCTGCGGCACGAGGAGGGGAGCAGTGAACGCGCTGTGCTCGGCGAAATGACCGTCGAGGACTTCTTGCCGAAAGCGGGCGCGGCTGCACAAGGCACGGATTCTGGTGTTGCGGACGCTCATGCAGTGCGTTCCCCCGGCCGAGGGAGCAATTCGTCGGCTGCTGCAGCGACCGCGCCGCCGCGTTCACGACGACGGAGGAGGGTAGCAACAGAGGACGTGGCCGAGAAGATGGTAGAGCGGAGGCAGAAGAGAATGATCAAGAACCGGGAGTCAGCTGCTCGATCTCGAGCAAGGAGGCAGGCTTACACGAACGAGCTAGAGAACAAGGTCGTCCTCCTCGAAGAAGAGAACCAAAGACTCGTGAAACACAAGGCAGAATTAGAAGCAGTAGAACGTAGCATGCCACATCCAGATCCGAAACACCAGCTCCGACGTACGAGTTCAGCTCCATTCTGA

**4.MaZIP101 （438 bp）**

ATGTCGCCTGCCCGGAGCCTCCAAGGTTCGTGGTCCGACGGAGACGCGAGGCTCACGGCAGAGGAGAGGAAGCAGAGGCGGAAGCTGTCGAACCGGGAGTCGGCGAGGAGGTCGCGGATTAGGAAGCAGCGGCAACTGGAGGATCTGACGAACCAGGTGGCCCAGCTGAGCAAGGAGAAGGGGCGGATCGTGATGCAGGTGGACGAGCTGGCGCAGCACCAGCTGCGGCTGGAGACGGAGAACGACATGCTGAGGGTCCGGGTGGCGGAGCTGACGGAGCGGTTGCGGTCTCTGAGCTCGGTGCTCCGCCTCGTGGAGGAGCTCAGTGGGGTGGCCATGGACGTGCCGGAGATTCCGGACCCGCTTCTCAAGCCGTGGCAGCCTCCCGGCCCCGCCTTGCCCGTCATGGCCGCCGCGGCGGACATGTTTCAGCCCTGA

**5.MaNCED-04 （1806 bp）**

ATGACTACTGCTACCAGTGCCATGACTTCTACTGTGCGATCAGCAGCCAATCCAGTGAAGCTGATACGCAAGCACGCCGCCCCTGCTACGCTAAGAATACGCTGCTCCGCCTCCAATTCGCTCCTTAACTTGACATCAGACGCCAGCCCCGCCTACTACCTGGCTCCTTGTCATAAGGAGGTGGCGCCAACTACCGTCGCGATGCCGGCGAGCAAGCCTGACCAGTCGACGCAAAGCTGCGGCGCGAGGCCGAGATGGAATTTGGTCCAGCGGATGGCCGCCGCGGCGCTAGACACGATCGAGGACGCGTTCGTGTCGAACGTGCTCGAGCGGCCGCGCCCTCTGCCGAAGACGGCGGACCCCGCCGTCCAGATCGCTGGAAATTTCGCCCCCGTCGACGAGCAGGCGCCGTGCCATGACCTCCCTGTTGAAGGCCGCATCCCGTCGTTCATCAGCGGGGTGTACGTCCGCAACGGCGCCAATCCTCTGTTTGAGCCTGTGGCGGGGCACCACTTCTTCGACGGCGACGGCATGGTGCACGCCGTCCACCTCCGCAACGGCGCCGCCACCTACGCCTGCCGCTATACCGAGACGGAGCGGCTCCGGCAGGAGCGCGCCATCGGGAAGCCGGTCTTCCCCAAGGCGATCGGCGAGCTCCATGGTCACTCGGGCATCGCGCGCCTGCTGCTCTTCTATGCCCGCAGCCTCTTCGGCCTCGTGAACGGCAGCCGCGGGATGGGCGTCGCCAACGCCGGCCTCGTGTACTTCAACGACCGCCTCCTCGCCATGTCCGAGGACGACATCCCCTACCACGTCCGCATCACTCCCTCCGGCGACCTCGAGACTGTCGAACGGTACGACTTCGGCGGACAGCTCCGCTCCTCCATGATCGCGCACCCGAAGCTGGACCCGTTTTCGCGCGAGCTCTTCGCGCTCAGCTACGACGTCATCCAGAAGCCTTACCTCAAGTATTTCTACTTCTCCCCTGACGGCAAGAAGTCCCCGGACGTGGAGATCCCCCTGGAACAGCCCACCATGATGCACGACTTCGCCATCACCGAGAACTACGTTGTGGTCCCGGACCAGCAGGTGGTGTTCAAGCTGCAGGAGATGATCCGCGGCGGCTCCCCGGTCGTCTACGACCAGGCCAAGACCGCCCGCTTCGGCGTGCTGCCCAAGTACGCCGCCGACGCTTCGGAGATGCGGTGGGTCGACGTGCCCGACTGCTTCTGCTTCCACCTGTGGAACGCGTGGGAGGAGCCGGCGACCGGCGAGGTGGTGGTGATTGGATCCTGCATGACGCCGCCGGACTCCGTGTTCAACGAATGCGAGGAGCGCCTCAACAGCGTCCTCTCCGAGATCCGGCTCGACCTCAACACCGGCAAGTCCACGCGCCGCTCCATTCTGTCCCCTGCCGATCAACTCAACCTCGAAGCCGGAATGGTGAACCGGAACATGCTGGGGCGGAAGACTCGGTACGCCTACCTGGCCATCGCCGAGCCATGGCCCAAGGTCTCGGGGTTCGCCAAGGTCGACCTCTTCACCGGCGAGATCAGCAAGTTCATCTTCGGCGACAGCCGGTACGGCGGCGAGCCCTACTTCCTGCCGCGCGATTCCAACTCGTTGAGGGAGGACGACGGCTACGTTCTCACCTTCATGCACGACGAGAAGACGTCCGCGTCGGAGCTACTGATCGTGAACGCCGTCGACATGCGACTCGAGGCCTCAGTCAAGCTGCCGTCGCGCGTTCCCTATGGCTTCCACGGGACCTTCGTCGGCTCAAAAGACTTGGAGTCACAGGCCTAG

**6.MaNCED-06 （1811 bp）**

ATGCTTTCTGCTGTCTGCAATGTTGCCAGTTCTTCTTCTTCCGTTGCAGTGTTGGGCCCGAGTAGGTCGAGATGCATTAAGATAACGAGAAAGCAATCCTTTACCGCTAGCTCAGTCCGTTGCTCCGCTTCCTTCGAGTCCCTCCCTGCTTCGCCACCACCTCCACCTTCTATCCGCCACTAGCGCATCTGACAGAGGACTACCCGTCGAGGCTGGAACGATCGAAACACCATGGCGATGACCGGATCATGAAACCCAGCAGGTGGAACATCTTCCAGCGGCTGGCTGCCGCAGCCTTGGACGGCGTCGAAGACGTCTTCATATCGAACGTACTCGAGCGCCGCCGGCCGCTTCCGAGGACCGCCGACCCTGCTGTTCAGATTGCTGGCAATTTCGGCCCGGTGGATGAGCGGCCTCCCTGCCGCAACCTTCCCGTCGATGGCCGGATCCCTGCCTCCCTCGACGGCGTGTACGTCCGAAATGGCGCCAACCCTCTATTCGAGCCTGTCGCCGGCCACCACTTCTTCGACGGTGATGGCATGATCCACGCCGTCCAAATCCGTGATGGCGCCGCTACGTATGCCTGCCGCTACACTGAGACGGAGCGCCTCCGGCAGGAGCGCGCCATCGGAAAGCCGGTGTTCCCCAAGGCCATTGGCGAGCTCCACGGCCACTCGGGCGTTGCCCGTCTCCTCCTCTTCATGGCTCGAGGCCTCTTCGGGCTCGTCGATCCCACGCATGGCACCGGGGTGGCCAACGCTGGCCTCGTCTACTTCAACGATCGCCTCCTCGCCATGTCGGAGGATGACGTCCCCTACCACGTTAGAATCACTCCCTCCGGTGACCTCGAGACGGTCGAGCGCTATGACTTCAACGGCCAGCTCTGCTCCTCCATGATCGCCCACCCGAAGCTCGATCCATCCACCGGCGAGTTGTTCGCTCTCTGCTACGACGTTGTCCGGAAGCCTTACCTCAAGTACTTCTACTTCTCTCCGGACGGCAAGAAGTCCCCCGACGTCGAGATCCCCCTCGAGCAGCCGACCATGATGCATGACTTCGCCATCACCGAGAACCACGTCGTGGTTCCGGACCAGCAGGTGGTGTTCAAGCTCCAGGAGATGATTCATGGCGGCTCTCCGGTCATCTACGACCGCGAGAAGATGGCACGTTTCGGAATTCTGCCCAAGCGCGCCCGCGACGCATCGGAGATGAAGTGGATCGACGTCCCCGATTGCTTCTGCTTCCACTTATGGAACGCGTGGGAGGAGCCGACGACCCACGAGGTGGTGGTCATCGGCTCTTGCATGACGCCGCCGGACTCTGTGTTCAACGACTGCGAAGAAAGCCTTCGAAGCGTACTCACGGAGATTCGGCTCAACCTCGCAACCGGAAAATCCACACGGCGCCCCATACTGTCATCGCAACTTAACCTCGAAGCCGGTATGGTGAACAGGAACAAGCTGGGGAGGAAGACCCGCTACGCCTACCTGGCCATCGCCGAGCCATGGCCCAAGGTGTCGGGGTTCGCCAAGGTCGACCTCTCCACAGGAGAAGTAAGCAAGTTCCTCTTCGGCGAGAGCAGGTACGGCGGCGAGCCTTGCTTCGTGCCAAGAAACGCGGGAGCGCTGTCGAGGGAGGACGATGGTTACGTCCTCACCTTCATGCACGACGAGAGAACGTCGGAGTCGGAGCTACTGATCGTGAATGCCGGCGACATGAGGCTCGAGGCAGCGGTGAGGTTGCCGTCCCGGGTTCCCTACGGCTTCCACGGCACCTTCGTGGCCTCCAAGGACTTGCAATCGCAGGCTTAG

**7.MaSDR-2（828 bp）**

ATGGCAACCTGCTCAGATCTGCCTGCCGACGCAAAGAAATTGGAAGGTAAGGTTGCCCTCATCACCGGTGGAGCCAGCGGCATCGGCGAGTGCACCGCCAAGCTCTTCTGCCGTCACGGAGCCAAGGTCGTCGTCGCCGACGTCCAGGACGAGCTCGGCACCGCAGTCTGCAGCGGTCTCGGCCCGGCCGCCTCCTTCATCCACTGCGACGTCACCAGCGAAGACGACGTAAGCGCCGCGGTCGACCACGCCGTCGCAAAGTTCGGCCGACTTGACATCATGTTCAACAACGCCGGCATCACCGGCGCCGCCTGCCATAACATCCTCCAGTGCGAGAAGTCCGACTTCGAGCGGGTGGTGGGCGTCAACCTGGTGGGGCCGTTCCTGGGGACGAAGCACGCGGCTCGCGTCATGATCCCGGCGCGCAAGGGGAGCATCATCACCACCTCGAGCCTGGCGGGGGTCATCGGCGCCGCCGCCGCGCACGCGTACACCTGCTCCAAGCACGCGGTGGTGGGGCTGACGAAGAACGCGGCGGCGGAGCTGGGCCAGTTCGGCGTCCGGGTGAACTGCGTGTCTCCTGCGGCGGCGGCGACGCCGCTGGCGATGAGATACGTGGGGCTCGAGGGCGAAGCCTTCGACATGGCCATGACCGCCGTCGCCAACCTCAAAGGGGTGAGCCTGGGGACGGAGGACGTGG

CCGATGCGGTGCTCTACTTGGCGAGCGACGACTCCAAGTACGTCAGTGGGCACAACCTTGTGCTCGATGGAGGATGCTCGGTGGCCTATCCTTCCTTCAATCTCTTTAAACGGGAGGAATCTCTTTAG

**8.MaAO (4182 bp)**

ATGGCGCGGAAGCTGGTGTTCGCCGTCAACGGAGAGCGTTTCGAGCTTGCCCAGGTCGATCCCTCCACCACCTTGCTCGAGTTCTTGAGGACCCAGACGCGGTTCAGAGGCCCCAAGCTCGGGTGTGGCGAGGGTGGCTGTGGAGCTTGTGTTGTTCTTCTCTCTACGTACCACCCTGTTAACGGGCAAGTGAAAGAATTTAGTATTAGCTCATGCCTGACGCTTCTTTGCAGCATAAATTTCTGTTCTGTTACCACCTCCGAGGGGCTGGGAAATAGTGAGGATGGCTTCCATCCGATTCACGAGAGGTTTGCAGGGTTTCATGCGTCCCAATGCGGATTTTGCACTCCTGGCATGTGTATGTCACTTTTTTCTGCTCTTACCAATGCTGACAAGACCAGTAGGCCTGAGCCTCCTGGTGGATTTTCGAAGATCACAAAGACCGAGGCTGAGAAGGCCATTGCCGGCAATCTTTGTCGATGCACTGGTTATCGGTCCATCGTAGATGTCTGCAAGAGCTTTGCAGCTAATGTCGATTTGGAGGACTTGGGCCTGAATACGTTCTGGAAGAAAGGGAATAAAGATGCAACGGTTTGTAGATTACCTCGTCATGGCCATAAAAGAATCTGCACATTTCCTGAGTTCTTGAAATCTGAGATCAAGTCCTCAATGGATATCTTGGACAATTTCAAGAATATGGGCTTGCCAGAGTGTCAGTGGTATCGACCTACCAGTATCGAAGAGCTTTATGAGCTTTTAAATTCCGACGCATTCCTTGAAAGCCATGTAAAATTGGTTGTTGGTAATACAGGGTCTGGTGTTTACAAGGAAAACGACCTGTATGATAAGTATATTGATCTCAAAGGGATTCCAGAGCTCTCGGTGATCAGAAGGGATAGTGGAGGGGTTTCATTTGGGGCTGCTGTGACGATATCTATGGCTATTGAAGTGCTGAAACAAAAAAATGAAAGTGAGCTGCACTCTAATGAAAGATTGGTCTTCAGCAAGATTGCTGATCATATGGATAAGGTGGCTACACCATTCATTAGGAATATGGCAAGCTTAGGAGGAAATTTAATTATGGCACAAAGAAGTCAATTTGCCTCAGATGTCGCTACAATACTTCTTGCTGCTGGATCAACTATCTGCCTTCAGACAGCTTCAGAAAGGCTAGTTCTTCCACTGGAGGAATTTTTACAAAGGCCTCCCTGTGATGACAGAACTGTACTTATAAACATACACATTCCTTTTTCAACTTCTGTAATGGAGTCATCTTCTGGAGCTAAAGGATGCATTGATTCTGAACCTACAAAGGAAGCCAATATACTATTTGAAACATACCGAGCAGCCCCACGACCTCTTGGAAATGCTATTGCTTATGTGAACTCTGCCTTTTTGGCTCATGTTACTTCATATAACATCTCTGGGGATCTTGTTATACATAATATACATTTGGCCTTTGGTGCTTACGGCAGTGAACATGCCGTGAGAGCAAGAAAAGTTGAGAACTTTTTGGTGGGTAAATCTGTCACTGCCTCTGTTTTACTTGGAGCCATTAAATTACTTAAGGAAACCATCATACCAAATGAACACACTCCTCATTCAAGATATAGATCAAGTTTAGCAATTGCTTTTCTATTCAAATTTTTTCAACCACTACTAAAAGACTTGAGTGTGCCTGAGAAGAACGTTCAAATGTCTGTTTCTAGTGCTGCTGCGACAATTGAAAACTCCAATGGCTGTATCAGTGGATTTGCTGATGATCTGCCTCGTAGGGCATCAAATGTTAAACAACTTGATCAAGCGAACAATCCTGATCTGATCTTATCTTCGGAGCAGATGGTTGAGTTTTGCAAGGATTATCATCCAGTTGGTGATCCTATCAAAAAAACTGGAGTTGAATTACAAGCCTCTGGTGAAGCAATATACGTGGATGATATTCCTTCTCCAAAGTACTGTCTTTATGGAGCATTTGTAAATAGCACAAGGCCTTTGGCACATATAAAGGGAATCAAATTCAAATCTACATCATCATCGCAGAAAGCCTTTACATTTATTGGTGCTGATGACATTCCAAAAGGAGGTCAAAATGTTGGATTATCTTGTCAATATGGAACTGAATCTTTATTTGCTCACTCTCTGACTGAGTGTGCTGGTCAGCCACTTGGCATTGTGATTGCAGAAACACAGAGACAAGCTAACATGGCTGCTAAACAAGCAGATGTACAATATTGCACTGAGAACTTAGAACCTCCAATTTTGTCAGTTGAAGATGCTGTTAGAAGATCCAGCTTTTTCAAAGTTCCTCCATTTTTGTGCCCTCAAAAGGTTGGAGATCTCTCCAAAGGAATGGCAGAAGCTGATCACAAGATTCTCTCAGCTGAGGTGAAGCTTGGTTCTCAGTATTATTTTTACATGGAAACACAAACAGCTCTTGCCATACCAGACGAAGACAATTGTATTTTGGTCTACACTTCAACTCAGTGTCCCGAGATTGCACAAGGTACTATTGCAAAATGCCTAGGCATACCTGCTCACAATGTCCGGGTTATTACAAGAAGAGTTGGTGGAGGTTTTGGTGGGAAAGGACCAAGATCAGTGCCTGTTGCGACTGCATGTGCTCTTGCAGCATTTAGATTGCGCCGTCCTGTTAGGATGTACCTGGATCGCAAGACAGATATGATAATGACAGGAGGGCGACATCCAATGCACATAAACTACTCTGTGGGTTTCAAGGCTGACGGGAAAATTACAGCCTTGCATGTAGATATCTTAGTCAATGCAGGAATAACAGCTGATGTTAGTATAATTATACCATGTAACATGGTATCAGCACTAAAGAAGTACAACTGGGGTGCTCTTTCTTTTGATATTAGGCTCTGCAAGACTAATTTTTCAACAAAATCAGCTATGCGGGGTCCAGGAGAAGTACAGGGAACTTTTATTGCTGAATCTGTTATTGAACATGTAGCATCATTCCTGTCCATTGATGTGAATTCTGTTCGAAAGAAAAATCTCCACACCTATGATAGTCTTATGTTGTATTATGAAGGTAGCACTGGAGATGCTCCTGAATATACTTTACCTACTATGATTGATGAGTTGGCTTCATCTGCTAGCTACTTTGATCGTCTTGAAATAATACGGCATTTCAATAGTTGCAATAAATGGAGAAAACGTGGAATTTCTTTGGTACCTGTTGTGTACCAAGTAGTGCTACGACCAACACCTGGGAAAGTATCTATTCTAACTGATGGTTCAATCGTTGTCGAAGTTGGAGGAATTGAGATTGGTCAGGGGCTGTGGACAAAGGTGAAGCAAATGACAGCATTTGCCCTTGGACAATTATGGGTTGATGGAAGTCAAAACCTTTTGGATAGGGTGCGGATCATTCAGGCAGATACTTTGAGTTTGGTTCAAGGAGGCTTGACTGCTGGGAGCACCACATCTGAAGCAAGTTGTGAGGCAGTTCGTCTATCCTGCAATGTTCTAGTTGATAGACTAAAGTCTCTAAAGCAAAGTTTAGAGGACAAAACAGGTTCTATCTCATGGGATACACTAATTTTTCAGGCAAATATGCAGTCTGTGAACTTGTCAGAGAGTACATACTGGGTTCCTGAAGATGCTTCTATTAGCTATCTCAATTTTGGAGCTGCTATAAGTGAGGTGGAGGTAGATGTTCTTACTGGAGCTACTATAATTTTGAGGACTGACCTTGTATATGATTGTGGACAAAGCTTGAATCCTGCTGTGGATTTGGGACAGATTGAAGGAGCATTTGTTCAAGGCATTGGTTTCTTCATGTGTGAAGAATACCTTGAGAACTCTGATGGCTTGGTGATTTCAGATGGTACTTGGACATACAAAATCCCAACTATTGACACCATCCCGAGGCAGTTCAATGTCAAATTATTGAACAGTGGACATCATGAAAAACGGGTTCTCTCATCGAAAGCTTCTGGGGAACCACCTTTAGTTCTGGCAAGTTCAATTCATTCCGCAACTAGGGAGGCCATAATAGCCGCTCGGATGGAATTCTCTTCTCCTACTGGCTCTGATAGCTCATCATCATTTCGATTGGAAGTGCCGGCAACCATGCCCGTGGTCAAGGAGCTCTGTGGCCTTGACAATGTGGAGAAGTACTTGAAAAACTTAGTGTCTTCCCATCAAGTGAAAGCCTAA
