# Supplementary material for: Overexpression of a Banana Aquaporin Gene MaPIP1;1 Enhances Tolerance to Multiple Abiotic Stresses in Transgenic Banana and Analysis of Its Interacting Transcription Factors
Source: Front Plant Sci. 2021 Aug 25;12:699230. doi: 10.3389/fpls.2021.699230 (PMC8424054; doi:10.3389/fpls.2021.699230)
Supplement: Supplementary file 7 [file Table_7.DOCX]

**Fig. S7**  Sequences of *MaMYB1R1*, *MaERF14/1/39*, *MaDREB1G*, *MabZIP53* and *MaMYB22*

1. **MaMYB1R1 (732 bp)**

ATGGTAAGGAAATGTTCTCACTGTGGACACAATGGCCACAACTCCAGGACTTGTATCAACACAAGAAGCAGCATGGTTGGTGGAGGTGGATGTCTCTCCTACACTTACCCTGCTGCTTCTTCCTCTTCCCCATCATCATCATCATCTTCTCTTGGTTCCATAGATGAAGCCACCCAGAAGATATCTCATGGTTATCTCTCTGATGGTCTTGGTGGCAGAACACAGGAGAGGAAAAAGGGAGTGCCATGGACCGAGGAAGAACACAGATCGTTTCTTATTGGACTCGAGAAACTTGGAAAGGGAGATTGGCGAGGCATCTCCCGCAACTTTGTGACCACAAGGACCCCAACACAGGTTGCTAGCCATGCTCAGAAGTACTTTCTCAGACAAAACAGCCTCAACAAGAAGAAGCGTCGATCGAGCCTTTTCGATGTGGTTGCAAACTGTGAGGGGGCAGCTCACAACACTGCCTCTTCGAAGTCAGACGATGCTTCCTTCTCATCGGAGCTGCATGCTCCGACTCTGTCCCTCACCATGGCAGGCCATAAAGCTTCTGAAACTACCACAATCGATCTCAACTCTTCAGGCCAAGAACAAGTAATACAGATGCCATCATCACAGTCACCTTCCTCCATGGAAAGGACTCGTGCTCAGCTCTCAAATCTGGATTTAGAGCTCAGGATTTCCTCATCCCCTGGCAGCCTATGTCTTGGAACCATCAGAGTTACTTAA

**2.MaERF14 (498 bp)**

ATGGTGAAGAGCAAGATCAGAGGGGCTCTCGATCACTGTGACCCAAGGAGCAGTGCTGCTGCAGTCAAAGGTAAGAAGCAGTACAAGGGTGTGAGGATGAGGAGCTGGGGCTCATGGGTCTCCGAGATCAGAGCACCAAACCAGAAGACCAGGATTTGGCTGGGCTCCTACTCCACGCCGGAGGCCGCGGCGCGAGCCTACGATGCGGCCTTGCTCTGCCTGAGAGGCACCGCCGCCAGCCTCAACTTCCCGGCCTCTCTGCTCCTGCAGCTCCCGGATCAAGTCATGTCGCCCAAGTCCATCCAAAGAGTGGCTGCCGCAGCTGCCACGAACGCTGCGTCGCCGTCGGATTCTCCCATCGATGACTCGATCAACTTCGAGGCCTTCTTCCAGTCACCCAAGTGTAGGGATTACATGCTTAACCCATCTCTCTTCTTTGCTCCTCAAGCCGAAGAGTGGGGTGAGGAAGCTGACATCCATCTGTGGAGCTTCTGCTGA

**3.MaERF1 (810 bp)**

ATGTGTGGAGGGGCGATCATCTCCAACATCATTCCGGCGGGCCCGAGGTCGCGACGGCGGACCGCCGGCTACCAGCGGCCGGACCCGAAGAATGAATTTGGAAAGAAGGACGACGACTTCGAGGCGGATTTCATGCAGTTCGAGGAGGACGAGGAGGCTGACCTCTTCCACTTCAAGCCCTTCGCTTTCGATTCGAGAGATAAACCAGTCCCTCTGAGGCCTTCAGTGATTGATAAACCTTCATCTAAGTCTGCTACAAAGAACAGAAAGAATCGATATAGGGGAATCCGCCGGCGTCCCTGGGGGAAATGGGCAGCTGAAATTAGAGATCCGTGTAAGGGAGTCCGTGTCTGGCTTGGAACCTTTAACACTGCTGAAGAAGCTGCCAGAGCATATGATGCTGAAGCTCGTAAGATCCGTGGCAAAAAAGCAAAGGTCAATTTCCCCGATGCAGCATTCCCCCGTGTCCGGACGCTCCTTACAAAACTGACTGCACCAGAAACTCCAAGAACAGAGATGCCCAGAACTGATAAGTGCTTCAATTACTTGAATGACCCATTTCAAGATTTCTGCTCTAGTTTTGACTTCACTGAAGTGGAGCCCATTATACAATCTGAGGAACTGACATACTTGGAAAATGGTGGTACTAAAAAACAACTGAATAACATTTTTTTCCAGCAACCTTATTCAGATGGGAGCACAGATGACTTGATTAACAACCTTCTCAGTGGTGATTTCACTCAAGATATGAGCAACGTGGATCTCTGGAGTTTTGATGGCATGGCACACATGGGAGGCAGTGTCTTCTGA

**4.MaERF39（519 bp）**

ATGAAGGCGCAGCCGACGACGGCGAGGTACAAGGGAGTGCGATTGAGGAAGTGGGGCAAATGGGTAGCCGAGGTGCGGTTCCCCAACAGCCGCCAGCGGCTGTGGCTCGGCTCCTACCCGACGCCCGAGATGGCTGCGCGAGCCTACGACGCCGCCGTGTACTGCCTGCGCGGCCCCGGCGCGGCGTTCAACTTCCCGAGCCACCCACCCAGCATACCGTCGGCCGACAAGCTGAGCCGGTACGAGATACGTGAGGCGGCCGAGCGGCACGCGCGCGAAGGGCCGCAGCGGGAGGAGGCAGAGGAAGCTGGCGAGCAAGTGGTGGATCCTGGTGCGGGAAGCTCTTGTTTAGGGGTGCCGTCGGGGCAGCCGGCCGAGCCGTCATCATCAGTGCCGGTTTTCGACGACGCCACAGCGAGCGGTGGGGAATGGTTCGATGGTTTCTGGTATGACGCCGGGGGCGGCAACGACGATGACGATATCTATCGATCGTCTCCTCTTTGGAACTTCCATCAGTAG

**5.MaDREB1G (636 bp)**

ATGGATAGCTTCAGCAGCGACTCGTTGGACTCGCCGTTGGCCCGGAGGTCGGCGGGGGCGGCGGCGTCGGACGAGGAGGTATCCTACGCGACGGTGTCATCGGCGCCGCCCAAGAGGCGGGCGGGGCGCACCAAGTTCCGGGAGACGCGGCACCCGGTGTACAAGGGGGTACGCCGGCGCAACGGGGACAAGTGGGTGTGCGAGGTGCGGGAGCCCAACAAGAAGTCCAGGATCTGGCTGGGCACCTTCCACACCGCCGAGATGGCCGCCCGGGCGCATGACGTGGCCGCCATGGCCCTGCGCGGCCGCTCCGCCTGCCTCAACTTCGCCGACTCCCCGTGGCGGCTCCCCGTGCCGGAGTCATCCAGCCCCGCCGACATCCGCAAGGCGGCGGCCCGCGTCGCCGAGGCCTTCCTGCCACGGCCGTCTTCCGAGACGCCCGAGCAAATGCAGGAGCAGATGGCGACGGCGTGGGCGGCAGCCGACGATGCCTTCTTCGTGGAGGACGGCCTCAACTTCGGAATGCAGGGGTACCTGGACATGGCAGAAGGTTTGTTGATCGATCCACCGCCGCCTCCGATGGACTACGAGGACGACAGCGACGGCATTGTTCCCCTGTGGAGCTATGCCGTCTGA

**6.MabZIP53（435 bp）**

ATGTCTTCCATTCCGGTCCGCCGCGCTTCGAGTTCTGAAGGAGACTCGCAGCCGACGTCCGATGAGAGGAAGAGAAAGAGGATGATTTCGAACAGGGAGTCCGCAAGAAGGTCTAGGATGAGGAAGCAGCAGCATCTCGATGATCTGATAAACCAAGCCGAGCAGCTCAAGAACCAAAACAGCCAGATCGACGTGCAGATCAATCTGGCGACACAGCAGTACGTCAAGGTGGAATCTGAGAACGCTATTCTAAGGGCTCAGCTGAGTGAATTGACAGAGAGACTGCACTCGATCAACTCTGTTCTCCGTTTCATTGAGGAGGTCAGTGGAATGGCCATGGACATACCGGAGATACCAGATCCTCTCCTGAAGCCATTGCAGCTTCCCCGTGCGGCACAACCAATCATGGCCAATGCTGACATGTTGCAGTTCTGA

**7.MaMYB22（900 bp）**

ATGACGACAAGGTCGTGGATGGAAGTCCTTCCTCCGGCGACGGTGCCGTGTTACCCCAGCTCAAGTTGGTTCATTGGCGAGAAAATGAGTGGGGGCGGCATCGGAGGCGGAAACTGGACCCCGGAAGAGAATAAGCGATTCGAGTATGCCCTGGCGAAGTTCGACAAGGACACCCCTGACCGCTGGGAACAGGTGGCGGCGTCTATCCCCGGTAAGACCGCGTGGGACGTGGAGAGCCACTACCGGGATTTGTTGGACGATGTGAGCGACATAGAAGCCGGGCGGATCCCATGTCCTGGCTACGACTCTTCGTCTTTTACACTGGACTGGGAGACCAATTACGGCTTCGAAGCGTCCACGCAACCTTACTGCATTGGTGGGAAGAGGTCAGCAGCGCGAGCATCGGATCAAGAGAGGAAGAAAGGAGTTCCCTGGACCGAAGATGAGCACAAGCGCTTTCTGTTTGGTCTCAAGAAATATGGAAAAGGGGATTGGAGAAATATATCTCGGAATTTTGTGATCACTAGAACCCCTACCCAAGTCGCTAGTCATGCACAAAAGTACTTCATCAGACTTAATTCAGGTGGCAAAGATAAGAGGAGGTCCAGCATACATGACATTACTACTGCCAATTTGCCTGATAATAGGCCTCCTTCTCCATCTCAGTCATCCGATCCTGCCACTCAGACAAGCTTGGCTTCTACACCGCTACCGTCGGCCCCATTCTCATCGATCCTTGATTCGAGTCATCCCAATGAAGCAACTACAATTGCAACTTCTTCGGTGCAGGGGAGTACAATTCGTGCAACCAAATTATGGAGTGACACCTTATGGTCTGCAACTAGAAGATCATGCACCTCGGAGTGGCACACTCGATGCTACCGTGGTTCAGGACCATGA
