# Supplementary material for: Overexpression of a Banana Aquaporin Gene MaPIP1;1 Enhances Tolerance to Multiple Abiotic Stresses in Transgenic Banana and Analysis of Its Interacting Transcription Factors
Source: Front Plant Sci. 2021 Aug 25;12:699230. doi: 10.3389/fpls.2021.699230 (PMC8424054; doi:10.3389/fpls.2021.699230)
Supplement: Supplementary file 13 [file Table_13.DOCX]

**Fig. S13** Sequence of *MaPIP1;1* gene promoter

CAAACATGGCGATCAAGGTAGCATTTGTCTGATATCAATCGAACGTTCTTGTTGATCGAATCAACCATCAATTCCACGAGGAAGACACGACACGAGACGACTCTGTCATCTGCACCGACAAATACACTAGTGTATTTGACGACGGCCAAACATTGCTATCAGTGGCCACTGTGACATGTGAGGCGGTCGAGTGTGACACGAAGTGGTAGGAATCCTATTTCACGTCCAATATATATATATATATATATATATATATATATATATATATATATATATATATTCAAAATATTTTAAAAAAAATTATAAATAATTATTTATTCTTTGCCTCGATCAATCTTGTTGTCGCTACCCCTATGCAGCCAGCTATGGCAATGATCTTATCAAATCCTTCCTTATGCAGAAAAGATACAAGCGAGGGAGGACGATAAAAGCTATCATCGCAACACCTCTTCTTTCCTTGCACCAGAACTGTCTCTACCTTCGTGAACCCCATCAAATCCTTTTTTGCGTGTAAAGCTTGTAAGATAAGGATAATAATGATTTACAAAGGTAATGTTAAGTCCAACGAGATAAAAAAAAAAAAGAGAGAGAGAGAGAGAGAGAGAGAGATAAAAATAAATTGATTGCGAGATGATCAATGAAATTTTTTAAAACTTAAAACGTTATCCAAGAATTTATCAAAATAATTTTAAAAAATCACCTATATACATATTAACCAGATAATTTGAATTAAATTTTAGAATTTAAAATATAAATCATTATGGCTACAGCCAACGTGGAATCCGTAGCCGTTGTCTGCGAATCCCAACTTTAAACGCACGTCCTCCCTTCGCTCCGCAAGGCCGCAACGGTCGTCTCACACTGGGAAGCGTTTCCGACGCAGCGAAGTTTCGTTCATCATCCCTGCTTTTGTCGTGTCCTCCCTCCTCCTCAGCTCACCCACGGGACGACGAAGAAGCCGGAAAGAGGAACGCAAACCGGCTTCGCCTCGAGCCGAGCGTCAGGAAAAGCTTTTGCCATTCTTAACCTCGACGTAGCCGAGCTGCTTTACGGAGCAGGTCAAAAAGCAAAAGCAGATAGATGGTGAAGAAAGAAGCCTCGAGGCCACAATTGCACGCCATGTTGCCATCAGGGAATCATCCCTATCTATCTTATTAGCACTGTACTCCATCTCCGATTAGTCTCGTGGCGAGACAGCACAACCCATCTGCTACCACGCCACCCCGCCTCTACGCCTATAAAGCCGCCAACAACACCCTGATTCCTCCACTCAC

GTGAGTGGAGGAATCAGGGT

ACGTG:bZIP-binding site TAACCA:MYB-binding site ACCGAC:DREB-binding site AGCCGCC:ERF-binding site
